# What are the effects of animals on the health and wellbeing of residents in care homes? A systematic review of the qualitative and quantitative evidence

**DOI:** 10.1186/s12877-023-03834-0

**Published:** 2023-03-25

**Authors:** Noreen Orr, Rebecca Abbott, Alison Bethel, Sarah Paviour, Rebecca Whear, Ruth Garside, Joanna Thompson Coon

**Affiliations:** 1 NIHR ARC South West Peninsula (PenARC), College of Medicine & Health, Evidence Synthesis Team, University of Exeter, South Cloisters, St. Luke’s Campus, Exeter, EX1 2LU Devon UK; 2 College of Medicine and Health, University of Exeter, Exeter, UK; 3 European Centre for Environment and Human Health, University of Exeter Medical School, Royal Cornwall Hospital, Knowledge Spa, Truro, Cornwall, TR1 3HD UK

**Keywords:** Animals, Companion animals, Animal therapy, Pets, Long-term care, Dementia, Systematic review, Wellbeing

## Abstract

**Background:**

There is some evidence to suggest that animal-assisted interventions can have beneficial impact for residents in long-term care, but the focus of the evidence has largely been on behavioural and psychosocial measured outcomes. Animals, either as companion animals or in the form of pet/animal-assisted therapy, may provide benefits in the form of social contact, as well as opportunities for sensory experiences and meaningful engagement not picked up by outcome tools. This review aimed to create a state-of-knowledge synthesis, bringing together qualitative and quantitative findings, on the impact of animal-human interaction on care home residents and care home staff.

**Methods:**

Fourteen databases were searched from inception to July 2020. Forward and backward citation chasing of included articles was conducted. Screening was undertaken independently by a team of reviewers. Thematic synthesis and meta-analysis were used to synthesise the qualitative and quantitative data.

**Results:**

Thirty-four studies, published in 40 articles (20 qualitative and 20 quantitative) were included. Five themes relating to resident wellbeing were identified in the qualitative evidence synthesis. These were animals as ‘living beings’, reminiscence and storytelling, caring (as ‘doing’ and ‘feeling’), respite (from loneliness, institutionalisation, and illness), and sensory engagement. A sixth theme related to staff perceptions and wellbeing, and a seventh to animal health and wellbeing. Maintaining identity was identified as an overarching theme. The majority of randomised trials had small sample sizes and were rated as low quality, mostly showing no evidence of beneficial effect. There was, however, limited evidence of a positive effect of pet/animal interaction on outcomes of loneliness, anxiety and depression, supporting the themes of respite and sensory engagement.

**Conclusions:**

The presence of animals can significantly impact the health and wellbeing of some care home residents. Residents had meaningful relationships with animals and derived pleasure and comfort from them. Interacting with animals offered residents a way to maintain a sense of self in the care homes, and with support, residents with dementia could also express their identities. Facilitating residents to interact with animals as part of person-centred care may also help residents to feel ‘at home’ in the care home.

**Trial registration:**

PROSPERO registration no: CRD42017058201.

**Supplementary Information:**

The online version contains supplementary material available at 10.1186/s12877-023-03834-0.

## Background

The number of older adults needing long-term care worldwide is on the increase. In the UK, in 2011 291,000 people aged 65 and over were living in care homes in England and Wales [1] and by 2020 this has risen to > 360,000 [2]. In the USA, latest estimates place over 1.3 million older adults living in long-term care [3] and similar rises are being seen throughout Europe [4]. In addition, people living with dementia comprise a large proportion of the resident population of care homes [5, 6]. Moving from independent living to residential/nursing care is a key transition in an older person’s life and can significantly affect an individual’s quality of life [7, 8]. A recent systematic review by Gardiner et al. suggested that as many as 61% of older people living in long-term care may be moderately lonely and around 35% may be severely lonely [9]. The recent restrictions on family visits and group activities as a result of COVID-19 infection measures has further highlighted the issue of social isolation and loneliness for those living in long-term care [10, 11].

A home-like environment is important to residents [12], and the presence of animals or pets may contribute to a feeling of less institutionalised living [13]. Companion animals and pet/animal-assisted therapy may provide people with access to different forms of social contact, as well as providing opportunities for sensory experiences and meaningful engagement. The systematic use of animals or pets in the context of therapy for the purpose of improved health and wellbeing is often referred to as animal-assisted intervention (AAI). AAI can encompass animal-assisted activities (AAAs), focussing on the motivational, educational and/or recreational benefits of animals or animal-assisted therapies (AATs), which tend to be more structured, goal orientated and planned and often led by a trained therapist [14]. Although AAA and AAT have separate definitions, there is often overlap between them in practice and studies do not always differentiate between them [15]. There may also be opportunity for human animal interaction from less organised activities in this setting, such as live-in pets and domestic animals kept by the home itself.

There has been a number of systematic reviews over the past 10 years assessing the impact of AAI on the health and wellbeing of older people in general [16–18]. Some have focused specifically on people living with dementia or cognitive impairment [19, 20] or have limited the review to dog specific AAIs [21–23]. The majority of reviews have had mixed findings and focused on quantitative outcomes. None to date have brought together qualitative and quantitative evidence relating to AAI specific to older adults living in long-term care. This review aims to create a state-of-knowledge synthesis on the impact of animal-human interaction on care home residents and care home staff. In particular we sought to answer:

1. what are the experiences, views and perceptions of residents, families/carers and care home staff of interacting with animals in older adult residential care settings? (*to be answered by the qualitative evidence*),
2. what are the measured effects of animals on the health and wellbeing of older people living in residential care and of the staff that care for them? (*to be answered by the quantitative evidence*),
3. are there different approaches or interventions (i.e. resident pets, pet visitation programmes, group or individual format, spontaneous or guided interactions, short- or long-term) that are particularly appropriate for different groups of residents (i.e. those living with dementia)? (*to be answered by both qualitative and quantitative evidence*),
4. what is known about the effects of human-animal interaction on the therapy/participating animal in care homes? (*to be answered by both qualitative and quantitative evidence*).

## Methods

Our review used best practice methods of evidence synthesis [24] and was developed in consultation with three relevant professionals (a care home owner, a care home manager, and a veterinarian) who formed our Expert Advisory Group (EAG). A full protocol outlining the methods of this systematic review which followed the Preferred Reporting Items for Systematic Reviews and Meta-Analyses (PRISMA) guidelines [25] was registered with International Prospective Register of Systematic Reviews (PROSPERO) CRD42017058201.

### Search strategy

The search strategy was developed by an information specialist (AB) and used a combination of relevant controlled vocabulary terms (e.g. MeSH) and free text terms. The original search was for robotic pets and visiting pets/animals which explains the search strategy for MEDLINE which is shown in Additional File 1: File S1 (and Table S1). The following databases were searched from inception to April 2017, update searches were run in July 2018 and again in July 2020: MEDLINE All from 1946, Embase from 1974, APA PsycINFO from 1806, SPP (via Ovid), CINAHL Complete, AgeLine from 1978 (EBSCOhost), CDSR, CENTRAL, DARE (Wiley Online, Cochrane Library), ASSIA (ProQuest), Web of Science Core Collection (SCI-Expanded, SSCI, A&HI, CPSI-S, CPSI-SSH, ESCI), SCOPUS and ProQuest Dissertations and Thesis Global with no date or language restrictions. Forward and backward citation chasing of each included article was performed.

### Study selection and eligibility criteria

Eligible articles had to report either i) the views, experiences and perceptions of animal interactions of older people resident in care homes, their families and carers and care home staff, or ii) the effects of animal interactions on health and wellbeing (including depression, agitation, loneliness and stress and quality of life), social interaction, engagement, physical function, behavioural symptoms, medication use and adverse events. Care homes was used as an encompassing term for residential care home, nursing home, and long-term care facility.

For studies describing views, experiences and perception, eligibility was restricted to qualitative studies using recognised methods of qualitative data collection (such as interviews, focus groups and observations) and of analysis (such as thematic analysis, grounded theory, and Interpretative Phenomenological Analysis). For studies reporting effectiveness, eligibility was limited to randomised controlled trials (RCTs), randomised cross-over trials and cluster randomised trials. Eligibility criteria were applied to all unique titles and abstracts by two researchers (RA, NO, SP or RW) independently. The full text of articles initially considered as meeting the inclusion criteria were retrieved and the eligibility criteria applied in the same way. Discrepancies at both stages were discussed and resolved with another reviewer (JTC) where necessary.

Data were collected using standardised, bespoke data extraction forms, piloted for use in this review. Data were extracted by one of four reviewers (NO, RA, RW and SP) and fully checked by another. Data extracted related to the study design, setting, participant characteristics, the human-animal interaction (animals involved, type of interaction, format, duration etc.), reported experiences and perceptions relating to the interaction and outcome measures.

### Quality appraisal and risk of bias

We used the Wallace criteria [26] and Cochrane Risk of Bias Tool [27] to critically appraise the qualitative and quantitative studies respectively. Qualitative studies were appraised by two reviewers (NO, RA). Quantitative risk of bias was performed by one reviewer (RA) and checked by a second (RW), with discrepancies discussed and resolved with a third (JTC).

### Data synthesis

We used thematic synthesis [28], an approach that draws on thematic analysis used in primary studies, to synthesise the qualitative studies. It is a three-stage process comprising coding of text line-by-line, identification of ‘descriptive’ themes and the development of ‘analytical’ themes. One reviewer (NO) coded lines of verbatim text labelled ‘results’ or ‘findings’ within the included studies. The text included participant accounts and author interpretations. Texts were coded to represent meanings inherent in the original manuscripts rather than to fit any pre-determined model or framework. Example codes included ‘animals created a positive atmosphere in the home’ and ‘petting animals helped residents to relax and calm down’. Groups of related codes were combined and systematically organised into descriptive themes. The descriptive themes were then re-interpreted inductively to develop analytical themes that, together, addressed review questions (i), (iii) and (iv). The codes and themes were examined and discussed a number of times among two reviewers (NO, RA), to ascertain similarities, differences, and connections between them [28]. We found that our discussions were aided by using a thematic network approach to structure and depict the themes (and the relationships between them) as a web-like network [29]. We arranged the descriptive themes into networks, grouped around the analytical themes, and then grouped these around a ‘global’ or ‘macro’ theme. The global theme, at the core of the thematic network, was the theme that summarised and interpreted the other themes within the network. As an illustrative tool, the thematic network emphasised the ‘fluidity’ of the themes and the ‘interconnectivity’ throughout the network [29]. The synthesis was guided by the ENTREQ (‘Enhancing transparency in reporting the synthesis of qualitative research)’ statement [30] (See Additional File 1: Table S2).

For the quantitative data, random effect meta-analyses were performed where we had sufficient data assessing the same outcome [31]. Pooling was performed on the outcomes measured immediately following the intervention. As we used a random-effects model for the meta-analyses, the weightings for each study were determined not only by the size of each study included, but also by between-study heterogeneity. Unadjusted summary data were used to calculate standardised mean differences (SMDs). As all the outcomes were continuous, pooled effects are reported as standardised mean differences with 95% confidence intervals. Where there were differences in the number of individuals contributing to baseline and follow-up summary statistics, we used the average sample size. Where data could not be pooled, a narrative summary was undertaken.

To bring the syntheses together, we adopted an interweaving approach [32]. We used deductive methods to draw together the findings within the qualitative and quantitative syntheses separately. We then sought to explore where the quantitative effectiveness data could help verify or contrast suggestions put forward by the qualitative data, and where qualitative experience data could help to explain why an intervention may be effective or not. The two lead authors (NO, RA) were immersed in the entirety of the evidence base and started the process of the overarching synthesis as the findings of the individual syntheses were developing. This method helps to highlight the similarities (and differences) in findings from the qualitative and quantitative evidence. To visually represent the overarching synthesis, we used the qualitative evidence synthesis as a framework and mapped where the quantitative evidence supported or refuted the data, an approach we have used previously [33].

## Results

**Fig. 1:**
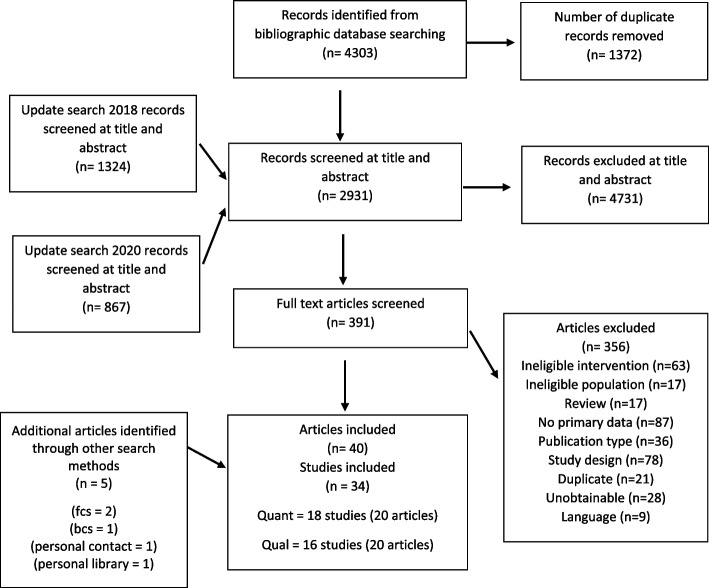
PRISMA

From the initial searches and update searches, 391 articles were selected for full-text review, resulting in 35 included full text articles. With an additional 5 articles found through supplementary searching, 40 articles (reporting on 34 studies) met the inclusion criteria (see PRISMA diagram in Fig. 1 for reasons for exclusion). The 34 studies included 16 qualitative studies and 18 randomised trials.

### General study characteristics

A summary of the main characteristics of all included articles is provided in Table 1.

**Table 1:** Study Characteristics

Author(s), year, country	Study aim(s)	Sample, size, characteristics	Intervention, duration, type, animal	Dementia focus?	Study design and methods *Analysis* (theory if mentioned)
Qualitative					
Casey (2018), *Canada *[34]	To explore the effects of an AAI program for residents with dementia on the work environment and perceived mental health and wellness of staff working with them	*n* = 20 staff (purposive) in a long-term care facility	AAI program comprised three 1 h sessions per week for 8 weeks. Residents engaged with a range of farm animals including a sheep, rabbits, chickens and a goat	Y	In-depth interviews with eight open ended questions. Recorded and transcribed verbatim *Thematic analysis*
Cook (2013), *UK *[35]	To evaluate the impact of the HENPOWER project on resident self-report of mental wellbeing, loneliness and depression	*n* = 21 care home residents, 1 relative, 22 staff, including managers (purposive)	HENPOWER programme aimed to establish henkeeping in six care homes	N	Focus group interviews and individual interviews. Recorded and transcribed verbatim *Thematic analysis* Realist Evaluation
Dookie (2013), *Canada *[36]	To explore the potential benefits of AAA on elders’ empowerment, self-esteem and quality of communication with caregivers in retirement homes	*n* = 10 residents who had a pet (purposive)	Residents with a personal pet living in five semiautonomous retirement homes	N	Semi-structured interviews. *Interpretative Content Analysis*
Fossey & Lawrence (2013), [37] *UK*	To identify the perceived advantages of involving animals in the life of the care home; staff concerns regarding the inclusion of animals; factors that facilitate their presence; and barriers to their inclusion	*n* = 108 staff from 15 care homes (purposive)	Staff with past and present experiences of a wide range of animals discussed the perceived benefits and difficulties associated with animals living at or visiting the care home	N	Fifteen focus groups. Data collection became progressively more focussed, and emerging themes were tested out in subsequent focus groups *Thematic analysis*
Freedman (2021), *UK *[38]	To explore what the experience of keeping a personal pet in a care home means for residents’ sense of wellbeing	*n* = 7 residents from four different care homes, three had dogs and four had cats (purposive)	Residents with a personal pet living in care homes	N	Semi-structured interviews with residents *Interpretative Phenomenological Analysis* Phenomenology
Gundersen & Johannessen (2018) , *Norway *[39]	To gain insight into the experiences and motivations of volunteer dog handlers and nurses who are involved in dog visits to nursing homes	*n* = 8 dog handlers, 10 nurses who had attended at least two dog visits (purposive)	AAA comprised dog visits at four nursing homes with groups of three to five residents in a suitable room and at least one employee (nurse or nursing assistant). The handlers planned and led the visits	Y	Individual in-depth interviews with the dog handlers and group interviews with the nurses *Inductive approach to data analysis.* Salutogenic theory
Kawamura (2009), *Japan *[40]	To examine how institutionalised older adults who have been participating in AAA for two years perceive the activity, and what relevance these perceptions have on clinical nursing practices in the AAA context	*n* = 8 residents (women) in a private nursing home living with dementia (no details given)	AAA sessions twice per month for two years. At each session three or four dogs were taken to the nursing home and the residents were able to freely feed, hold and play with the dogs for approximately 30 min	Y	Semi-structured interviews (approximately 30 to 50 min). Interviews were recorded and transcribed. *Colaizzi’s phenomenological methodology for data analysis* Phenomenology
Kendziorski (1999), *USA *[41]	To determine the effects of resident pets on residents living at a local nursing home, including perceived quality of life, and examine the relationship between the residents and the resident animals	*n* = 10 residents from one nursing home, all previous pet owners) (purposive)	Informal interaction with the resident animals. Primary resident pet – Golden Retriever, also resident birds and fish	N	Semi-structured interviews following the hermeneutic process *Hermeneutic analysis for data analysis*
Kongable (1990), *USA *[42]	To evaluate nursing staff feelings and attitudes about the use of a pet dog as a therapeutic agent for residents residing in a special care Alzheimer’s unit	*n* = 6 nursing staff associated with the pet therapy programme	Programme involved the use of pet therapy. The dog was brought to the unit for three hours, one day per week	Y	Interview with five open-ended questions (approximately 15 min) *Content analysis*
Pitheckoff (2018), *USA *[43]	To obtain insider knowledge of the experience of participating in an existing animal-assisted activity program that provides older adults with an opportunity to interact with rabbits	*n* = ~ 30 residents (observed) and 8 residents (interviewed)	AAA (Rabbit assisted activities). Activity typically involved three rabbits that were informally shared with residents in their rooms and in common areas. Activity session lasted for one hour	N	Direct observation of two activity sessions conducted several weeks apart Eight face-to-face, in-depth, semi-structured interviews (generally lasted 45 min) *Thematic Analysis (Triangulation of observation data and interview data)*
Pooley (2007), *UK *[44]	To produce an explanatory model to improve understanding of the psychological benefits of pet ownership by investigating the significance of companion animals for older people who move with them into homes for older people	*n* = 9 residents (living in nursing, residential and sheltered housing, 7 managers (purposive)	Residents with a personal pet living in care homes	N	Semi-structured interviews with residents; managers of the homes; observations in the homes were conducted in the communal areas (one hour) *Grounded theory methodology* *Grounded theory*
Roenke & Mulligan (1998), *USA *[45]	Explore the qualitative aspects of pet therapy in order to better understand what it is about such experiences that is perceived as beneficial from the participant’s perspective	*n* = 4 residents, 1 pet therapy volunteer (purposive)	Two sessions of pet therapy (one with a five month old kitten, and one with two eight week old kittens)	N	Participant observation of two sessions of pet therapy; group interviews of three residents, individual interview with one resident, individual interview with pet therapy volunteer. Interviews consisted mainly of open-ended questions *Thematic analysis*
Savishinsky (1985), *USA *[46]	Describe the results of a long-term, intensive research project conducted on the Cornell Companion Animals Program (CCAP), a university-based, volunteer organisation	Residents in three nursing homes (*n* = 72, 75 and 250). Approx 10% -30% resident involvement	Volunteers conducted weekly visits using either their own pets or borrowed animals offered to CCAP. In the smaller homes, pet visitation was in a group format, in the larger home, individual residents met with pets in their own rooms or lounge areas	N	Direct participant-observation, unstructured interviewing and life history work. Researcher also completed participant observation on non-pet related recreational activities at the three homes *No details on data analysis*
Swall et al. (2015), *Sweden *[47]	To illuminate the meaning of the lived experience of encounters with a therapy dog for persons with Alzheimer’s disease	*n* = 5 residents living in a nursing home with medium to severe Alzheimer’s Disease	AAT – Ten visits per person with therapy dog. Prescription is individualised with a specific justification for each planned visit, for example, to increase alertness or to decrease anxiety	Y	Video observations The first step of the analysis was a naïve reading, followed by structural analysis and a comprehensive understanding *Phenomenological hermeneutics* Phenomenology
Swall et al. (2016), *Sweden *[48]	To illuminate meanings of the lived experiences of dog handlers’ when visiting older persons with dementia with their therapy dog	*n* = 9 dog handlers	As above	Y	Interviews with open-ended questions (as above) *Phenomenological hermeneutics* Phenomenology
Swall et al. (2017), *Sweden *[49]	To illuminate meanings of care for people with Alzheimer’s Disease in their encounters with a therapy dog	*n* = 5 residents with medium to severe dementia	As above	Y	Video observations focusing on the interaction between the person with AD and the therapy dog (as above) *Phenomenological hermeneutics* Phenomenology
Swall et al. (2019), *Sweden *[50]	To describe the impact of therapy dogs on people with dementia in the final stages of life from the perspective of the dog handler	*n* = 7 dog handlers (convenience)	As above	Y	Semi-structured interviews Participants were asked to talk about situations when they had visited people with dementia with their therapy dog *Qualitative content analysis*
Wong (2015) *New Zealand *[51]	To examine the experience of animal therapy in two residential aged care homes that receive animal visits from an animal welfare organisation	*n* = 7 residential aged care home residents (convenience)	AAT visiting programme involving puppies, kittens, adult dogs, rabbits and guinea pigs, rats and adult cats. Visits were either once a week or once a fortnight and each visit lasted approximately an hour	N	In-depth, semi-structured interviews *Narrative analysis* Narrative gerontology
Wong & Breheny (2021), *New Zealand *[52]	To examine the experience of animal therapy in two residential aged care homes that receive animal visits from an animal welfare organisation	*n* = 7 residential aged care home residents (convenience)	AAT visiting programme involving puppies, kittens, adult dogs, rabbits and guinea pigs, rats and adult cats. Visits were either once a week or once a fortnight and each visit lasted approximately an hour	N	In-depth, semi-structured interviews *Narrative analysis* Narrative gerontology
Zando (2017), *USA *[53]	To explore how residents diagnosed with Alzheimer’s disease respond to contact with a certified therapy dog	*n* = 5 nursing home residents (purposive)	Researcher met with each resident in privacy of own room, introduced the therapy dog and engaged the resident in a conversation about the dog. There were three visits and each visit lasted for no more than 15 min	Y	Recorded observations of physical and/or verbal responses the resident had to the dog. Observations were coded to identify significant themes, categories and patterns. A consensus process with peer colleagues was used to support the identification of themes
*Quantitative*					
Andrysco (1982), *USA *[54]	To determine the effectiveness of animal assisted therapy in a nursing home and explore appropriate methods for assessing resident-animal interaction	*n* = 46 residents in 1 long-term care home. Age range 70–99 yrs	Individualised AAT. Residents received 1 × 20 min session with a dog over 10 weeks. Control group had a visit without a dog	N	RCT. Primary outcome was interaction and behaviour (assessed by questionnaire). Secondary outcome social interaction (assessed by video). Analysis by (3-way ANOVA)
Banks et al. (2008), *USA *[55]	To compare the ability of a living dog and robotic dog (AIBO) to treat loneliness in resident living in long-term care	*n* = 42 residents in 1 care home. No age metrics – described as elderly	Individualised AAT. AAT groups received weekly visits lasting 30 min from either a dog robot (AIBO) or a living dog for 8 weeks	N	RCT. Primary outcomes of loneliness (UCLA questionnaire) and attachment (Lexington attachment to Pets Scale). Means were compared by analysis of variance
Banks (1998; 2002), *USA *[56]	To determine whether AAT is effective in combating loneliness among elderly adults	*N* = 15 residents in 3 long-term care facilities (70% > 75 yrs)	Individualised AAT. AAT groups received either weekly or thrice weekly visits lasting 30 min with a dog. The control group received no visits	N	RCT. Primary outcomes of loneliness (UCLA-LS questionnaire) and pet history questionnaire also completed. Analysis using ANCOVA
Bumsted (1988), *USA *[57]	To investigate whether pet therapy has a favourable effect on resident self care	*n* = 20 from 1 large nursing home. Mean age 83yrs (I), 86 yrs I	Individualised AAT. Residents received 6 × 25 min sessions over 3 weeks with a dog. Control group had no visits	N	RCT. Primary outcomes were self-care and physical self-maintenance (Bespoke questionnaires). Analysis by MANOVA
Briones (2021), *Spain *[58]	To assess the effectiveness of AAT on quality of life in residents with dementia in a care home	*n* = 39 residents with dementia in 1 care home. Mean age 88yrs(I) 89yrsI	Group AAT. Residents had 1 × 50 min session per week for 9 months. Control had usual care, no visits	Y	RCT. Primary outcomes was quality of life (QOL-AD). Functional status also assessed (Barthel Index). Analysis using ANCOVA
Colombo (2006), *Italy *[59]	To determine whether pet therapy had a favourable effect on psychopathological status and on perceived quality of life	*n* = 144 from 7 nursing homes. Mean age 78 yrs (I and C)	Companion pet. Residents either had a canary or plant to look after in their room, or usual care for 3 months	N	RCT. Primary outcome was quality of life (LEIPAD-short version). Brief symptom inventory (BSI) also completed. Analysis by variance analysis
Friedman (2015), *USA *[60]	To evaluate the use of structured activities with a dog (PAL intervention), to prevent deterioration of physical function, emotional and behavioural function	*n* = 40 residents with dementia from 7 long-term assisted living facilities. Mean age 80 yrs (I) 82yrs I	Group AAT. Residents had 2 × 60–90 min session per week with a dog over 12 weeks. Control group had reminiscence sessions	Y	CLUSTER RCT. Primary outcomes of activities of daily living (Barthel Index) and depression (CSDD) and agitation (CMAI). Analysis using linear mixed models
Greer (2002), *USA *[61]	To examine the effect of live cats compared to toy cats on the verbal communication of individuals with dementia	*n* = 6 residents in 1 nursing home with Alzheimer’s disease. Age range 84 -90yrs	Group AAA. Residents (groups of 3) had 3 × 10 min session with 2 live cats. Control phase involved 2 toy cats	Y	CROSSOVER RCT. Primary outcome was the number of words spoked (observation). Analysis approach not specified
Johnson (1997), *USA *[62]	To determine whether pet encounter therapy (PET) has a favourable effect on mood states and social facilitation	*n* = 100 residents in 6 skilled nursing facilities. Mean age 85yrs (I) and 78yrs I	Individual AAT. Residents received either an animal (rabbit/kitten), stuffed toy, or person only visit of 10 min. Control had no visit	N	RCT. Primary outcome was affective mood (MAACL). Analysis using ANOVA
Le Roux (2009), *South Africa *[63]	To explore the effect of AAA on the depression and anxiety levels of residents in a long-term care facility	*n* = 16 residents from 1 home. All > 65 yrs	Group AAA. Residents spent time with a dog for 1 × 30 min, over 6 weeks. Control group had no visits	N	RCT. Primary outcomes were depression (BDI) and anxiety (BAI). Analysis using Wilcoxon signed rank test
Olsen (2016), *Norway *[64]	To examine the effects of AAA on depression, agitation and QoL in nursing home residents with dementia or cognitive impairment	*n* = 58 residents from 10 nursing homes. Mean age 83yrs (I) and 84yrs (C)	Group AAA. Residents received 2 × 30 min dog visits per week, for 12 weeks. Control group had no visits	Y	CLUSTER RCT. Primary outcomes were depression (CSDD), agitation (BARS) and quality of life (QUALID). Analysis using ANOVA
Panzer-Kaplow (2000), *USA *[65]	To investigate the effects of AAT on levels of depression and morale among residents of a geriatric nursing facility	*n* = 35 residents from 1 nursing home. Mean age 72yrs (I) and 73yrs (C)	Individual AAT. Residents received 1 × 15 min dog visit per week, for 10 weeks. Control group received no visits	N	RCT (Matched pairs). Primary outcomes were depression (BDI &GDS) and morale (PGCM). Analysis using t-tests
Pope (2016), *USA *[66]	To compare the effectiveness of AAT versus human interaction only on social behaviours and engagement among elderly patients with dementia in a long-term care facility	*n* = 44 residents from 1 skilled nursing facility. Mean age 80yrs	Individual AAT. Residents had 2 × 10 min/week sessions for 2 weeks with a dog, Control phase involved visits with human only	Y	CROSSOVER RCT. Primary outcome was social behaviour (observation) and engagement (MPES). Analysis using ANOVA
Thodberg (2016a & b), *Denmark *[67, 68]	To explore the effects of a real animal (dog) compared to robot/ toy animals on the psychiatric wellbeing and sleep of residents with dementia	*n* = 124 residents from 4 nursing homes. Overall mean age 85yrs	Individual AAT. Residents received 2 × 10 min visits with a dog, robotic seal or stuffed toy cat,	Y	RCT. Primary outcomes were behaviour (GBS), sleep quality, depression (GDS) and cognitive functioning level(MMSE). Analysis by linear regression
Travers (2013), *Australia *[69]	To assess the effect of dog-assisted therapy for people with dementia living in aged care facilities on mood and quality of life	*N* = 67 residents from 3 aged care facilities. Mean age 85yrs (I and C)	Group AAT. Residents received 2 × 40/50 min sessions a week, for 11 weeks with a dog. Control group had therapist visit only	Y	RCT. Primary outcomes were depression (GDS), quality of life (QOL-AD) and psychosocial functioning (MOSES). Analysis by linear regression
Valenti Soler (2015), *Spain *[70]	To compare the effectiveness of AAT (dog) versus therapeutic sessions with a robotic animal (PARO) or usual care for residents with moderate to severe dementia	*n* = 110 residents from 1 nursing home. Mean age for all 85yrs	Individual AAT. Residents received 2 × 30-40 min sessions per week for 12 weeks with a dog or robotic seal. Control group had usual care	Y	RCT. Primary outcomes were behaviour changes (NPI), apathy (APADEM-NH) and quality of life (QUALID). Analysis with a mix of non-parametric tests
Wall (1994), *USA *[71]	To explore whether a purposeful goal orientated interaction with a companion animal facilitates positive changes in residents wellbeing	*n* = 80 residents from 11 skilled nursing facilities. Mean age 88yrs(I) and 83yrs(C)	Individual AAT. Residents received 3 × 6-10 min sessions with a dog over 3 weeks. Comparator groups (stuffed toy or human)	N	RCT. Primary outcomes were speech activity (audio) and mood (MSE). Analysis by ANOVA
Zulauf (1987), *USA *[72]	To explore the effects of a dog-facilitated therapy visitation program on psychosocial wellbeing	*N* = 40 residents from 1 nursing home. Age range all: 60-92yrs	Individual AAT. Residents received 1 × 30 min visit/ week with a dog for 6 weeks. Control group human visit	N	RCT. Primary outcomes were depression (GDS), Morale (PGCM) and self esteem (SES). Analysis by ANoVA

*AAI* Animal-assisted intervention, *AAA* Animal-assisted activities, AAT Animal-assisted therapy, *UCLA* University of California Los Angeles loneliness Scale, *BSI* Brief symptom inventory, *MAACL-R* Multiple affect adjective checklist – revised, *BDI* Beck depression inventory, *BAI* Beck anxiety inventory, *BARS* Brief agitation rating scale, *CMAI* Cohen-mansfield agitation inventory, *CSDD* Cornell scale for symptoms of depression in dementia, *GDS* Geriatric depression scale, *MOSES* Multidimensional observational scale for elderly subjects, *MS-E* Mood scales – elderly, *SES* Self esteem scale, *QUALID* Quality of life in late-stage dementia, *QOL-AD* Quality of life in alzheimer’s disease, *AES* Apathy evaluation scale, *NPI* Neuropsychiatric inventory, *APADEM-NH* Apathy scale for institutionalized patients with dementia nursing home version, *NOSIE* Nurse observation scale for inpatient evaluation, *RAID* Rating anxiety in dementia, *PGCM* Philadelphia geriatric center morale scale, *MPES* Menorah park engagement scale

### *Qualitative studies*:

In total twenty qualitative papers (from 16 studies) were included in this review. Studies were carried out in the USA (*n* = 6), the UK (*n* = 4), Canada (*n* = 2), and one in each from Norway [39], New Zealand [51, 52], Japan [40] and Sweden [47–50]. Studies reported on the experience of a wide range of human animal interactions including: residential home pets and animals kept on site (e.g. chickens) [35, 41], personal pets of the residents [36, 38, 44], and animal assisted interventions such as dog therapy or small animal visits (e.g. kittens, rabbits) (*n* = 10). One study considered staff members’ experiences of a wide range of animals living at, or visiting, care homes [37]. Eleven of the studies had a primary focus on the effects and experiences from resident perspectives, three focussed on the experiences and perceptions of care home staff [34, 37, 42], and three studies (across six papers) explored multiple perspectives (e.g., experiences and motivations of the dog handlers, nursing staff attending therapy sessions and/or the residents). Five of the studies focussed on the effects/experiences for residents living with dementia [40, 47, 49, 50, 53].

### *Quantitative studies*:

Twenty papers, reporting on 18 randomised trials were included in this review. Interventions were described as pet encounter therapy, pet-facilitated therapy, pet-assisted living, animal assisted intervention, animal assisted therapy, animal assisted activity or simply dog visits/therapy. The majority of studies were from the USA (*n* = 11), and the remainder were conducted in Norway [64], Italy [59], South Africa [63], Australia [69], Spain [58, 70] and Denmark [67, 68]. Nine of the studies had a specific focus on residents living with dementia [58, 60, 61, 64, 66–70]. The sample size of the studies were generally small, ranging from six [61] to 144 residents [59], with eleven of the studies involving less than 50 residents.

Fifteen of the studies involved dogs as the intervention, one study involved cats [61], one study assessed in-room canaries [59], and one study reported on the effect of kitten and rabbit visits [62]. Intervention duration varied from a one-off visit [61, 62], to interventions of three to six weeks [57, 63, 66, 67, 71–73], longer interventions of 8–12 weeks [54, 55, 59, 60, 64, 65, 69, 70] and in one study, nine months [58]. The approach of the intervention was either one-to-one [54, 55, 57, 58, 62, 65–68, 70–73], including one study in which the animals (canaries) lived with the residents in their room [59], or was group–based [58, 60, 61, 63, 64, 69]. Touching of, and interaction with, the animal was reported as actively encouraged in seven of the studies [59, 64–67, 69, 70]. There was only one quantitative study that involved resident animals [59], with the remainder assessing the impact of visiting animals.

### Study quality

#### Qualitative studies

All of the studies had a clear research question, used appropriate study designs and adequately described how the data were collected. The sample was drawn from the appropriate population in all of the studies but was considered adequate in just over half of them; ethical approval and gaining informed consent from the sample were noted in most studies. Just over half of the studies noted a theoretical framework which influenced the study design. In a small number of studies, it was difficult to appraise data collection and/or analysis due to inadequate reporting [34, 41, 42, 44–46, 53]. Most of the reported findings were substantiated by the data shown (See Additional File 1: Table S3).

#### Quantitative studies

Overall the quality of the randomised trials was poor, see Additional File 1: Table S4 for a summary of the risk of bias across the studies. Less than half of the trials were assessed as being at a low risk of bias for random sequence generation, and only three studies reported on methods that demonstrated adequate allocation concealment [64, 67, 70]. Almost all studies performed poorly in terms of the blinding of participants and personnel involved, with only one study clearly reporting methods that suggest a low risk of bias for outcome measurement blinding [70]. All but one trial [68] was assessed as low risk on selective reporting, and all but two trials [54, 62] were assessed as a low risk of bias on reporting of outcome data. There was a high proportion of items in ‘other bias’ rated as unclear due to the presence of sizable gaps in reported information for the domains of whether the appropriate analyses were used and whether the baseline data were equal across groups prior to the intervention starting.

#### Qualitative synthesis

Five analytical themes were identified relating to resident wellbeing: animals as ‘living beings’, reminiscence and storytelling, caring (as ‘doing’ and ‘feeling’), respite (from loneliness, institutionalisation, and illness), and sensory engagement. The theme ‘respite’ was informed by Swall et al.’s study [48] and was broadened to encompass respite from loneliness and institutionalisation. A sixth theme related to staff perceptions and wellbeing, and a seventh to animal health and wellbeing. All of these themes grouped around the global theme of identity (see Additional File 1: Figure S1 for the complete network of identified themes).

In this section we present the seven themes and Table 2 shows which studies contributed to each theme. Examples of quotations to support the themes are in Table S5 (Additional File 1: Table S5). The global theme of identity brings the themes together and offers an interpretation which is the focus of the discussion section.

**Table 2:** Summary of overarching themes

**Authors**	**Animals as living beings**		**Reminiscence and stories**		**Caring**			**Respite**					
					**Resident animals** **Being responsible, meaningful activity**		**Visiting animals** **Being affectionate, being concerned**	**From loneliness**		**From institutional-isation**	**From symptoms of illness**	**From pain and anxiety [at end of life]**	
Casey et al. (2018) [34]							✓				✓		
Cook et al. (2013) [35]			✓		✓			✓		✓	✓		
Dookie (2013) [36]					✓								
Fossey & Lawrence (2013) [37]					✓			✓					
Freedman et al. (2021) [38]	✓		✓		✓			✓		✓			
Gundersen & Johannessen (2018) [39]			✓					✓		✓	✓		
Kawamura (2009) [40]			✓				✓	✓		✓			
Kendziorski (1999) [41]			✓		✓					✓			
Kongable et al. (1990) [42]			✓				✓						
Pitheckoff et al. (2018) [43]	✓		✓					✓		✓			
Pooley (2007) [44]	✓		✓		✓			✓					
Roenke & Mulligan (1998) [45]	✓		✓				✓	✓		✓			
Savishinsky (1985) [46]	✓		✓					✓		✓			
Swall et al. (2015) [47]	✓		✓				✓	✓			✓		
Swall et al. (2016) [48]			✓				✓	✓			✓	✓	
Swall et al. (2017) [49]			✓				✓	✓			✓		
Swall et al. (2019) [50]												✓	
Wong (2015) [51]								✓		✓			
Wong & Breheny (2021) [52]							✓	✓		✓			
Zando (2017) [53]											✓		
**Authors**	**Sensory experience**				**Staff experiences**							**Animal**	
	**Physical contact/ touching**	**Watching**	**Pleasure and joy**	**Calmness**	**Enhanced workplace**	**Seeing residents engage**	**Better caring culture**	**Increase workload**	**Distraction from care**	**Health, hygiene & safety concerns**	**Management support & organisation**	**Positive**	**Negative**
Casey et al. (2018) [34]	✓		✓	✓	✓	✓	✓		✓				✓
Cook et al. (2013) [35]	✓	✓	✓	✓				✓	✓	✓	✓		
Dookie (2013) [36]													
Fossey & Lawrence (2013) [37]		✓	✓			✓		✓	✓	✓	✓		✓
Freedman et al. (2020) [38]													
Gundersen & Johannessen (2018) [39]	✓		✓	✓			✓				✓	✓	
Kawamura (2009) [40]			✓										
Kendziorski (1999) [41]			✓										
Kongable et al. (1990) [42]	✓	✓	✓								✓		✓
Pitheckoff et al. (2018) [43]	✓		✓	✓									
Pooley (2007) [44]						✓							
Roenke & Mulligan (1998) [45]	✓												
Savishinsky (1985) [46]	✓		✓									✓	
Swall et al. (2015) [47]	✓		✓	✓									
Swall et al. (2016) [48]			✓	✓			✓						
Swall et al. (2017) [49]	✓		✓	✓								✓	
Swall et al. (2019) [50]	✓			✓									
Wong (2015) [51]	✓		✓										✓
Wong & Breheny (2021) [52]	✓		✓										✓
Zando (2017) [53]	✓		✓										

#### Theme 1. Human-animal interaction – animals as ‘living beings’

Several studies highlighted that residents appreciated the special qualities of either their pet or visiting animal, or animals more generally [38, 45, 46]. In their discussions, residents attributed animals with ‘almost human’ qualities such as an ability to ‘know’ and ‘understand’. Authors believed that the ‘human’ quality or ‘human likeness’ of animals helped to create a bond between the animal and the resident, and intensified residents’ level of engagement ([45], p.39). Residents described how their pets could empathise with them and provide emotional support; authors suggested that this sense of support may be due to the ability of a ‘living being’ to respond to individual residents ([43], p.7). The responsiveness of animals to residents meant that some residents preferred engaging with their pet or visiting animal above other activities and other people [38, 47]. Pets could act as substitutes for human relationships: for example, one resident spoke of how she could ‘build a relationship’ with her cat and she described her as ‘family’ and a ‘best friend’ ([38], pp.1973–4). In some cases, residents spoke of animals as being almost superior to humans, praising their care for their young, their kindness, love and loyalty, and how they could teach humans to care and be kind [45, 46]. Comparing the behaviour of animals and people allowed residents to demonstrate ‘human frailties’: “I love animals so much (especially cats) because they don’t talk back” ([46], p.118). However, not all residents seemed to appreciate the special qualities of animals or their pets, as illustrated by Mrs Thomas: “I mean, she’s just a cat” ([44], p.8).

#### Theme 2: Reminiscence and stories of past pets/family/occupation

How animals helped residents reminisce was highlighted by many studies [38–49]. Reminiscing through stories of past pets, interwoven with stories of childhood and adulthood, was a way of connecting to past selves and maintaining a sense of self and identity. Interacting with visiting animals facilitated the recollection of past events and experiences with their pets or other animals. Memories of previously owned pets could emerge after prompting but there was also ‘spontaneous reminiscing’, from those living with dementia [42, 43, 46, 47, 49]. Residents shared both happy and sad stories: they recalled how animals could be a source of ‘domestic comedy’ and recounted humorous episodes from their childhood [35, 43]. For some residents, particularly those from rural backgrounds, reminiscing about pets was a stimulus to share stories of their experiences of working with, and caring for, farm animals [35]. Residents shared their sadness and feelings of loss when recalling past pets [47] and these feelings could influence how they responded to involvement with visiting animals: in one case a resident declined, explaining that animals were associated with sad memories of his past pets [46]. Also, for those living with dementia, memories of the visiting dog and dogs from their past lives could “in the long run create confusion” ([48], p.2227): “[s]witching between joyful and difficult—sad memories seemed to create uncertainty and fear over what was real and true” ([47], p.87). Past pets could often be a ‘connecting thread’ to a person they had lost such as a deceased spouse [44–46]. One resident explained that he had lost both his wife and his third Airedale dog at around the same time and that his current dog was a way of connecting with both [38]. The losses associated with living in a care home could be distressing and, as described by one resident, pets could, to some degree, compensate for losses experienced: “It takes the place of the people that have died” ([44], p.8).

For some residents, being a pet owner was an ‘enduring aspect of self’ and they could not “…remember a time when they had been without a pet or imagine a situation in the future when their pet might be absent” ([38], p.1969). Bringing their pets with them to the care home was an important factor in their choice of care home [38]. Those who had been unable to bring their pets with them spoke of how difficult it was to leave their pet—who they considered as part of their family—and how their pets had been given to family members or friends. One resident said, “…my cat…Princess…My daughter’s got her now…I hated to get rid of her, I’ll tell ya. But, you can’t do nothing about it” ([41], p.32). Visiting animals could prompt residents to talk about these pets and tell stories of how they came to live in the care home. These stories could be a mixture of regret about having to give up a pet and gratitude for the person taking care of it. Savishinky (1985) observed that some residents described their pets as being ‘on loan’ and that they expected to be reunited with them: “…pets are thus a way for such individuals to voice their perception of nursing home life as a temporary situation” ([46], p.125). Sometimes these stories also conveyed a subliminal message of feeling abandoned by family, as with one resident who had praised her family for taking on her pet beagle but then observed that “they kept the dog and got rid of me” ([46], pp.125–6). The current health and condition of their pets could be an important topic of conversation for residents and also suggests that pets were a ‘stimulus for contact’ between the residents and those caring for their pets.

#### Theme 3: Caring

### Resident pets – being responsible and meaningful activity

Residents demonstrated their care for resident and visiting animals in different ways and according to their interests and abilities. For those residents who were living with their personal pets, caring was regarded as a personal responsibility that often satisfied their need to ‘care for something’ [36, 38]. Caring for their pet could also be a way of maintaining the role of carer and keeping that “part of themselves that they valued alive” ([44], p.8). In some cases, residents needed staff help with the practical care of their pets, and accepted that sharing responsibility with others meant that the pet was less dependent on them [38]. Caring for a personal pet in a care home could lead to various worries and concerns for residents such as the pet running away (particularly on moving into the care home) and overfeeding of the pet without their knowledge [44]. Communal animals in care homes offered caring opportunities for those residents who were able and wanted to engage in a range of tasks such as feeding, grooming, cleaning, and collecting eggs [35, 37, 41]. Caring for animals in these ways could provide residents with meaningful activities and “…a sense of purpose and a routine that was different to that of tasks and personal care that so dominated their lives as care home residents”([35], p.62). However, some residents were clear about not wanting caring responsibilities for resident animals on a daily basis due to their physical disabilities [41]. There were also examples in one study which showed that caring could be detrimental for both resident animals, in particular overfeeding, and for residents who cared ‘too much’ and found it difficult to adjust to life without the pet [41].

### Visiting animals – feeling involved, affectionate and concerned

Residents could show their care for visiting animals through how they interacted with them and how they expressed their feelings of care for the animals. Caring seemed to be closely linked to having regular contact with a ‘known’ animal and having a perceived relationship with it [40, 52]. In one nursing home where three dogs visited over two years, residents developed a one-to-one relationship with the dogs, and gained ‘confidence’ in their ‘familiarity’ with them. Although they were not involved in their daily care, they indicated that they felt they had ‘close relationships’ with them and perceived themselves as having ‘a special role in the dogs’ lives’ ([40], p.46). The ‘developing familiarity’ with a particular animal could be ‘bidirectional’ ([52], p.2649), with residents getting to know the animal and the animal getting to know the resident. However, not all residents felt able to develop a relationship with, and care for, a visiting animal [45, 52] for various reasons—because the animal was not theirs, the brevity of the visits and the possibility of different animals on each visit.

Interestingly, living with dementia did not necessarily preclude residents from showing care for visiting animals: in one study, residents received visits from a therapy dog team and provided care through touch and gestures such as slow patting and putting their arms around the dog, and in conversation with and about the dog [47–49]. Residents were observed being affectionate towards the dog, “treating it as a precious living creature” ([49], p.4), and being concerned about any health problems that the dog may have had. However, residents could unintentionally mishandle animals “due to their diminished cognitive capacities” ([34], p.1245) and in one case, a resident became too possessive with the dog and attempted to pick it up to keep it away from other residents [42].

#### Theme 4: Respite

Animals could offer respite to residents in a number of ways: respite from loneliness; respite from institutionalisation; respite from the symptoms of illness; and relief from pain and anxiety at the end of life.

### Respite from loneliness

Animals provided comfort and companionship for some residents, and was particularly important for those who had brought their pets to the care homes and regarded them as the one aspect of their lives that remained constant [38, 44]. Their pets seemed to counteract their loneliness and helped ease the transition to living in a care home. Pets also helped their owners make friends with other residents and staff, and residents appreciated that others liked and enjoyed their pets, and the care that staff showed towards their pets. This, in turn, could help residents feel that they were well cared for themselves [38, 44]. A shared interest in animals enabled some residents to build relationships with staff, and animals and their antics could be a’talking point’ that staff and residents ‘enjoyed together’ [35, 37, 40]. Similarly, the presence of animals could lead to enhanced interactions between residents themselves and a greater interest in each other [39, 40, 43]. However, not all residents had opportunities to socialise with each other while experiencing the animal visits with some being confined to their beds and rooms [51, 52]. There was also the possibility that residents could display jealousy towards each other during animal visits [48].

Animals could also facilitate social encounters with people from ‘outside the care home’ such as volunteers [40, 45, 46] who could be volunteering with animal welfare organisations [52], or trained animal handlers [39, 47, 49]. For example, the HENPOWER project was set up in care homes by older community volunteers who assumed henkeeeping roles and interacted with residents, important for those who felt “isolated and cut off from the world” ([35], p.70). Dog handlers recognised the importance of having ‘small talks’ with residents using “the dog as a medium for contact and dialogue” ([39], p.108). For many of the residents, the social encounters with volunteers and animal handlers could become at least as important as the companionship provided by the animals [39, 46, 52], and in some homes, the regular presence of volunteers and pets helped create a ‘family atmosphere’ [46].

### Respite from institutionalisation

The presence of animals was described by a number of authors as creating a positive atmosphere and bringing an interesting dimension to the daily life and routines of the care homes [35, 38–40]. Residents reported seeing the resident dog daily [41], and in the HENPOWER project, hens were regarded by many as a positive addition to the care home, transforming the gardens into interesting places with residents reporting that they spent more time in the gardens [35]. Savishinky ([46], p.127) suggests that animals in care homes can ‘momentarily’ recreate ‘past domesticity’ in which residents can participate: “…pets are symbolic and literal embodiments of the more complete domestic experience that residents once had.” Anticipation of visits could break the monotony of care home routines and particularly, where there was a regular visiting schedule, residents expected the pets and looked forward to the visits [38, 45, 51, 52]. In one study, looking forward to the dogs visiting was described as the “most pleasurable part of their life in the nursing home” ([40], p.44). Some residents wished that the animal visits were more frequent or lasted longer [38, 43, 52], and reflected that the benefit was restricted to the ‘moment of interaction’, described by one resident as a ‘fleeting’ pleasure ([52], p.2650). Having regular visits with the same animal could help residents to maintain a ‘sense of reality’ ([45], p.37), and dog handlers also recognised that regular visits with a stable group of participants was important in helping them get to know the residents [39].

### Respite from symptoms of illness

A few studies illustrated that the presence of animals offered residents, particularly those with dementia, respite from the symptoms of their illness [34, 35, 39, 47–49, 53]. According to Swall et al. (2017), engaging with the dog (and its handler), could enable or empower some residents to ‘step out of the shadows’ of dementia and distance themselves from its symptoms, show something of their previous abilities and in that moment, act like a ‘healthy’ or ‘whole’ human being. Being with a dog could give residents a ‘temporary presence of mind’ ([47], p.87) or ‘episodes of lucidity’ [74], which could then uncover memories of childhood, nature and animals. The encounters with animals were often described as ‘moments’ (e.g. moments of joy and calm) which would not last; Swall et al. (2016) observed that the symptoms of the illness would return a few weeks after the visits stopped. Some staff could see the value of creating ‘good moments’ despite the likelihood that many residents would not remember the visits, which according to Casey et al. (2018: p.1245), meant that “each encounter [was] in essence a first encounter” and therefore, did not lead to any ‘sustained gains’.

### Relief from pain and anxiety [at end of life]

One study found that dog therapy could provide relief from pain and anxiety for people with dementia at the end of life [48, 50]. The dog handlers observed that the presence of the dog with its warm body had a calming effect, reducing hyperventilation and anxiety, as well as pain. They believed that the physical warmth of the dog and its calming presence helped residents ‘open up’ and talk about their imminent death with the dog and sometimes, with the handler too. It could also shift the focus from their situation of nearing the end of life to the dog and its wellbeing: one woman on her last night was concerned about the dog’s comfort, requesting that the window was shut so that it would not be cold. The dogs could also have a physical effect on the person such as when a man approaching death opened his eyes and started to pat the dog. The connection between the dog and the person in these moments was, as noted by the handlers, often because “the person and the dog had a history of visits together” ([50], p.68). However, the presence of a dog did not alleviate anxiety for all residents and the handlers noted that anxiety could be so strong in some, that they often ‘closed themselves off’ ([50], p.69).

#### Theme 5: Sensory engagement

### Physical contact with, and watching, animals brought pleasure, joy and calmness

Many studies emphasised how the presence of animals offered residents a sensory experience, primarily by tactile and visual stimulation [34, 35, 37, 39–43, 45–47, 49–53]. Watching animals or physical contact with an animal and its body through stroking, petting and cuddling could generate both verbal and non-verbal responses from residents, and often acted as a ‘source of diversion’ [42]. Staff, animal handlers and volunteers alike, commented on how physical contact with animals elicited positive responses from residents, and in some cases triggered memories and conversation. Through sensory engagement with animals many residents, including those with dementia, gained pleasure and joy, and also a sense of calm and comfort [49]. Residents spoke of how physical affection was key to their enjoyment and in one study, residents preferred a resident dog over fish and birds because they were able to physically interact with the dog [41]. Staff noted the importance of animals providing residents with non-judgemental and unconditional love [42]. The physical contact with animals seemed to induce calmness and provide relief from anxiety, important for those residents who may have been agitated [34, 48], and particularly, for those at the end of life [50].

#### Theme 6: Staff

Three studies reported staff interest and enjoyment in visiting animals and on the ‘positive feeling’ created within the care homes [34, 37, 39]. Staff understood that animals could make a difference to the residents—particularly if they had ‘long been a part of their lives’ ([37], p.317)—promoting interaction and engagement, and decreasing responsive behaviours for those living with dementia [34, 39, 44, 48]. Some staff believed that the animal visits made their jobs easier, helping them deliver person-centred care with a smaller number of residents while others were occupied with the animals [34]. Staff also appreciated the work of the dog handlers [39] and the person-centred care they offered residents, which arguably, contributed to a more caring culture within the care homes [37, 48].

There were staff that felt the presence of animals was disruptive to the routines of the care home and could distract them from caring for the residents. This mostly related to the perceived additional work that resident pets would generate such as cleaning and feeding, and staff reluctance to accept these tasks opened up the potential for animal neglect [37]. Staff could also lack confidence in their abilities and knowledge to care for animals as was the case with the hens in the HENPOWER project [35]. That animals could jeopardise care home hygiene was a significant issue for staff; other issues included the potential safety hazards of residents falling over animals and residents being allergic to animals. Importantly, staff recognised that communal animals could have a negative impact on those residents “at risk of being disturbed or distressed by their presence” ([37], pp.324–5).

Some staff regarded animals visits as a ‘waste of time’ and were overwhelmingly negative [34]; often the successful involvement of animals in care homes depended upon the “enthusiasm and responsibility of individual staff members” ([37], p .323–4). A supportive care home manager with a positive attitude and who was willing to manage risk appropriately was also very important [35, 39]. Staff identified a lack of clarity about policy and practice in relation to communal animals living in care homes, and a lack of guidance about how to arrange or conduct animal visits [37]. Clearly, care home managers should lead in developing a care home policy about animals, and in the planning and discussion with staff about job roles and responsibilities for introducing, monitoring and caring of animals [37, 42]. A clear procedure for assessing animal suitability for the home, and procedures and processes to deal with the problems that arise from introducing animals into care home settings such as infection control was also identified as crucial. Good organisation was also valued by the dog handlers when they visited care homes and it “…mattered…that they felt expected at the nursing home and that the nurses and residents were prepared for the visits” ([39], pp.108–9).

#### Theme 7: Animal wellbeing

We found little evidence in the studies about the effects of human-animal interaction on the wellbeing of the animals [34, 37, 42, 47]. The dog handlers in one study believed that the dogs appreciated the visits to the care homes and were committed to ‘the task’: “a wagging tail showed that they were eager ‘to go to work’” ([39], p.106). One volunteer described how the interactions with residents had helped her dog recover from depression and in her view, “…pet sessions [could] be as therapeutic for animals as for the people they visit” ([46], p.122). The degree to which some visiting animals benefitted from their interactions with care home residents may also have been influenced by their training and preparation. Two studies reported on how the dogs and dog handlers used in the care homes received training (18 months in Sweden) and were tested for suitability before being certified [39, 47]. In contrast, the visiting animals in another study were brought from an animal welfare organisation, primarily to socialise the animals in preparation for adoption, and there was no formal procedure for determining the suitability of the animal [51, 52]. This had the potential for causing fear and stress to an animal due to volunteers’ lack of knowledge about it and its behaviour [75]. Clearly, not all animals are temperamentally suited to the care home setting and not all are suited to the role of visiting animal [76, 77].

### Quantitative synthesis

Outcomes were grouped into three categories: psychological, behavioural and wellbeing. A detailed summary of the quantitative outcome results is shown in Table 3. Meta-analyses were only possible for the psychological outcomes of depression and anxiety, the behavioural outcome of agitation, and the wellbeing outcome of quality of life (see Additional File 1: Figure S2). A reduction in anxiety was the only significant outcome from pooled analyses. A narrative synthesis of the findings for comparable outcomes not able to be pooled and reported by more than one study is provided below.

**Table 3:** Summary of key outcome data from the randomised studies

**Theme** Study ID	**Outcome**	**Scale/Tool**	**Comparator groups and sample size** Intervention(I), Control (C), Other (Oth)	**Post Intervention scores or Change scores** Mean (SD)	**Interpretation and significance (as reported by authors)**
*Psychological well-being*					
Banks 2008 [55]	Loneliness	UCLA	AAT vs AIBO vs usual care (control) (I:13, Oth:12, C:13)	No data available	Control group statistically different from the AIBO (*P* < .05), and the AAT (*P* < .05) but no statistically significant difference between the AIBO and AAT groups
Banks 98/2002 [56]	Loneliness	UCLA-LS	AAT vs usual care (control) (I:30, C:15)	AAT1 40.56 vs AAT2 39.08 vs 48.7	Both AAT groups differed significantly from the control group (*p* < .01) but not from each other
Colombo 2006 [59]	Depression	BSI	Resident pet vs plant vs usual care (nothing to care for – control) (I:48, P:43, C:53)	0.79 (0.50) vs 1.10 (0.68) vs 1.40 (0.88)	Resident pet differed significantly to control and plant group (*P* < .001)
	Anxiety	BSI		0.78 (0.61) vs 0.71 (0.65) vs 0.92 (0.67)	Resident pet differed significantly to plant group (*p* < .05) but not to control
	Depression and anxiety	LEIPAD-SV		1.93 (1.94) vs 3.37 (2.13) vs 3.53 (3.08)	Resident pet differed significantly to plant group (*p* < .001) and control group (*p* < ..01)
Friedman 2015 [60]	Depression	CSDD	PAL vs reminisence (control) (I:32, C:28	5.21 (SE 0.77) vs 8.76 (SE 1.51)	No significant differences between groups (*p* = 0.07)
Johnson 1997 [62]	Depression	MAACL-R	PET vs toy vs human vs usual care (control) (I:17, toy: 16, Human:14, C:25)	0.43 (0.35) vs 0.72 (1.21) vs 0.57 (0.94) vs 0.94 (1.36)	No significant differences between groups
	Anxiety	MAACL-R		0.25 (0.46) vs 0.44 (0.65) vs 0.71 (0.91) vs 0.96 (1.11)	No significant differences between groups
	Positive affect	MAACL-R		3.25 (2.82) vs 2.50 (2.26) vs 3.66 (3.33) vs 2.91 (2.27)	No significant differences between groups
Le Roux 2009 [63]	Depression	BDI	AAA vs usual care (no visits – control) (I:7, C:8)	11.86 (8.75) vs 15.88 (10.18)	No significant differences between groups
	Anxiety	BAI		10.71 (7.61) vs 13.50 (10.73)	No significant differences between groups
Olsen 2016 [64]	Depression	CSDD	AAA vs usual care (control) (I:22, C:25)	7.86 (4.42) vs 8.28 (5.62)	More participants in AAA improved than in control group (*p* = 0.03)
Panzer 2000 [65]	Depression	**GDS** (+ BDI)	AAT vs usual care (control) (I:16, C:19)	Mean difference -2.44 vs -1.21	No significant differences between groups (*p* = 0.34)
	Morale	PGC Morale Scale		Mean difference 1.06 vs 0.37	No significant differences between groups (*p* = 0.44)
Thodberg 2016a [67]	Depression	GDS	AAT vs robot seal vs toy cat (I:35, Seal: 35, C: 30)	No data available	No significant differences between groups, but depression did improve over time for all groups
Travers 2013 [69]	Depression	GDS	AAT vs usual care (therapist only—control) (I:27, C:28)	4.0 (2.9) vs 2.6 (2.1)	No significant differences between groups
	Irritability	MOSES		10.0 (3.6) vs 11.1 (3.9)	No significant differences between groups
Wall 1994 [71]	Depression	MS-E	AAT vs toy vs Human only vs no visits (control) (I:20, Toy:20, Human:20, C:20)	1.38 (0.75) vs 1.38 (0.61) vs 1.65 (0.74) vs 1.91 (1.0)	No significant differences between groups
	Tense/irritable	MS-E		1.40 (0.56) vs 1.51 (0.69) vs 1.64 (0.56) vs 1.76 (0.66)	No significant differences between groups
Zulauf 1987 [72]	Depression	GDS	AAT vs Human only (control) (I:18, C:9)	AAT1 15.33, AAT2 7.39, C 12.39	No significant differences between groups (*p* = .05)
	Self-esteem	SES		No data available	No significant differences between groups (*p* = .05)
	Morale	PGC Morale Scale		No data available	No significant differences between groups
*Behaviour*					
Andrysco 1982 [54]	Behaviour	Direct and video observation	Pet therapy vs human only visit (control) (I:23, C:23)	No data available	Social interactions with other residents, activity involvement showed no significant differences between groups but dependency on staff did
Bumsted 1988 [57]	Selfcare	Self care agency tool	Pet therapy vs usual care (control) (I:10, C:10)	22.10 (8.20) vs 22.90 (8.75)	No significant differences between groups
		Physical self-maintenance scale		18.00 (7.82) vs 18.30 (6.65)	No significant differences between groups
Colombo 2006 [59]	Self care	LEIPAD-SV	Resident pet vs plant vs usual care (nothing to care for – control) (I:48, P:43, C:53)	5.45 (3.78) vs 3.25 (2.45) vs 7.36 (3.74)	Resident pet differed significantly to plant group (*p* < .01) and control group (*p* < .05)
	Social functioning	LEIPAD-SV		3.06 (1.52) vs 4.15 (1.95) vs 4.15 (1.95)	Resident pet differed significantly to plant group and control group (*p* < .01)
Friedman 2015 [60]	Apathy	Apathy evaluation scale	PAL vs reminisence (control) (I:32, C:28)	17.53 (SE 0.90) vs 15,72 (SE 0.82)	No significant difference between groups
	Agitation	CMAI		15.53 (SE 0.68) vs 20.00 (SE 1.69)	No significant difference between groups
Olsen 2016 [64]	Agitation	BARS	AAA vs usual care (control) (I:24, C:26)	23.75 (7.13) vs 24.65 (13.95)	No significant difference between groups
Pope 2016 [66]	Agitation	CMAI	AAT vs usual care (human only visit—control) (I:44, C:44 -crossover)	34.0 (12.8) vs 36.6 (13.4)	No significant difference between groups
	Social behaviour	Social behaviour checklist		157.08 vs 111.09	Significant difference between groups (*p* < .001)
Travers 2013 [69]	Self care	MOSES	AAT vs usual care (therapist only—control) (I:27, C:28)	17.5 (6.5) vs 17.4 (6.1)	No significant differences between groups
Thodberg 2016a [67]	Sleep	Acti-watch	AAT vs robot seal vs toy cat (I:35, Seal: 35, C: 30)	No data available	No significant differences between groups
Valenti Soler 2015 [70]	Behaviour	NPI	AAT vs usual care (control) (I:36, C:32)	22.33 (14.67) vs 28.66 (19.08)	No significant differences between groups (*p* = 0.65)
	Apathy	APADEM-NH		No data available	No significant differences between groups
Zulauf [72]	Behaviour	NOSIE (3)	AAT vs Human only (control) (I:18, C:9)	AAT1 87.17, AAT2 100.57, C 85.78	Significant differences between AAT2 compared with AAT1 and control (*p* < .05)
*Quality Of Life (QoL)*					
Briones 2021 [58]	Quality of life	QOL-AD	AAT vs usual care (control) (I:16, C:18)	32.46(1.27) vs 31.5(1.41)	Both groups improved from baseline, no significant difference between AAT and control
Olsen 2016 [64]	Quality of life	QUALID	AAA vs usual care (control) (I:24, C:26)	24.8 (5.79) vs 25.3 (10.26)	No effect of AAA on QoL at Post intervention *p* = 0.344 (or at follow-up *p* = 0.136 at 3 months)
Travers 2013 [69]	Quality of life	QOL-AD	AAT vs usual care (therapist only—control) (I:27, C:28)	A 34.0 (7.2) vs 38.9 (5.9) B 38.1 (4.4) vs 33.2 (5.3) C 34.7 (4.9) vs 39.6 (6.1)	Mean QOL-AD score in AAT was significantly higher (better) than in the control group (*p* = 0.02) in Facility B but was significantly lower (*p* = 0.02) in Facility C however an outbreak of Gastroenteritis during the final week of intervention in this facility may have influenced this
Valenti Soler 2015 [70]	Quality of life	QUALID	AAT vs usual care (control) (I:36, C:32)	24.33 (6.68) vs 24.72 (6.68)	No effect of AAT on QoL at Post intervention *p* = 0.101
*Engagement/Interaction*					
Andrysco 1982 [54]	Smiles	Direct and video observation	Pet therapy vs human only visit (control) (I:23, C:23)	No data available	Paired t-test showed no significant difference between the two groups though residents smiled more in the AAT group
	Verbalisation	Direct Observation			Overall improvement in verbalisation in intervention group but no significant differences between groups
	Eye contact	Direct Observation			Eye contact with researcher decreased during AAT as resident watched the animal. Differences not significant
	Tactile	Direct Observation			Residents did not touch researcher but wee touching animal 40–70% of the time
	Number of questions	Direct Observation			Residents asked significantly more questions about all topics during pet interactions
Greer 2002 [61]	Words spoken	Video observation	AAA vs toy cat (I:6, C:6 ABACA design)	24.8 words/min vs 19.3 words/min	Average total words were greater during the live cat intervention than in the toy cat intervention. Words/min continued to increase even after withdrawal in the live cat condition
	Meaningful information units	Video observation		6.2MIU/min vs 4.7MIU/min	The groups reacted differently in the withdrawal period
	Initiations	Video observation		2.5/min vs 2.1/min	Average initiations were greater during the live cat intervention than in the toy cat intervention
Johnson 1997 [62]	Smiles	Observation checklist	PET vs usual care (control) (I:25, C:25)	7 (3.25) vs 4.27 (3.47)	Significant difference between groups *p* < 0.02
	Verbalisation	Audio-recording		No data available	No difference between groups *p* = 0.67
	Eye contact	Observation checklist		7.65 vs 7.86	No significant difference between groups
Pope 2016 [66]	Engagement	Menorah Park Engagement Scale	AAT vs usual care (human only visit—control) (I:44, C:44 -crossover)	Mixed results across not engaged, self, passive and constructive engagement	No significant difference between groups *p* = 0.26
Thodberg 2016b [68]	Interaction	Direct observation (bespoke tool)	AAT vs robot seal vs toy cat (I:35, Seal: 35, C: 30)	Conversation (various) Physical contact (various) Eye contact (various)	Physical contact more likely with animal (*p* < .001) or seal (*p* = 0.01) than toy cat. Conversation more likely with animal or seal (both *p* < .05) than toy cat but there were several moderators. Eye contact more likely with animal than seal or toy cat over time
Wall 1994 [71]	Speech	Audio observation	AAT vs toy vs Human only vs no visits (control) (I:20, Toy:20, Human:20, C:20)	84.45 (35.07) vs 77.63 (38.05) vs 83.82 (39.05) (no data for control)	No significant differences between groups
Zulauf 1987[72]	Activity participation	Direct observation	AAT vs Human only (control) (I:18, C:9)	68.11 vs 59.00	Mixed results as AAT1 and 2 differed. No significant differences between groups *p* = 0.08

*UCLA* University of California Los Angeles loneliness scale, *BSI* Brief symptom inventory, *MAACL-R* Multiple affect adjective checklist – revised, *BDI* Beck depression inventory, *BAI* Beck anxiety inventory, *BARS* Brief agitation rating scale, *CMAI* Cohen-mansfield agitation inventory, *CSDD* Cornell scale for symptoms of depression in dementia, *GDS* Geriatric depression scale, *MOSES* Multidimensional observational scale for elderly subjects, *MS-E* Mood scales – elderly, *SES* Self esteem scale, *QUALID* Quality of life in late-stage dementia, *QOL-AD* Quality of life in alzheimer’s disease, *AES* Apathy evaluation scale, *NPI* Neuropsychiatric Inventory, *APADEM-NH* Apathy scale for institutionalized patients with dementia nursing home version, *NOSIE* Nurse observation scale for inpatient evaluation, *RAID* Rating anxiety in dementia

#### Psychological

### Depression and anxiety

Ten RCTs assessed the effect of pet therapy on depression [59, 60, 62–65, 67, 69, 71, 72]. The majority of the studies involved therapy/activity sessions with dogs, and sessions ranged from as little as 5-10 min/week to 2 X 90 min /week and occurred over a range of 3 to 12 weeks. One study involved assessing the effect of canaries that lived in the residents room [59], and another study looked at one off therapy sessions with kittens or rabbits [62]. Seven of the interventions were based on one-to-one interaction, and three used a group-based approach. Eight different validated depression assessment tools were used (see Table 3). Seven of the studies provided data that could be pooled for meta-analysis, involving a total of 173 residents in the intervention and 187 in the control groups. The pooled standardised mean difference (SMD) of effect on depression was 0.34 (95%CI -0.73 to 0.04; *p* = 0.08; I^2^ = 67%) (see Additional File 1: Figure S2). The three studies not able to be included in the pooled analysis individually reported no effect on resident depression.

Three RCTs reported on the effect of pet therapy on resident anxiety [59, 62, 63]. All used a one-to-one approach, and involved looking after a caged canary for three months [59], one-off sessions with kittens or rabbits [62] or a dog visit once a week for 6 weeks [63]. Anxiety was assessed using three different tools. The pooled results across the three trials, involving 158 residents, indicated evidence of a small reduction in anxiety, SMD -0.36 (-0.68 to -0.04, *p* = 0.03; I^2^ = 5%), see Additional File 1: Figure S2.

### Loneliness

Two RCTs, run over 6–8 weeks, assessed the effect of dog visits on resident loneliness [56, 73]. Both studies had usual care (i.e. no visits from a dog) as the control group. Banks et al. (2008) compared the effects of a real dog with a robotic dog, against the control group and Banks (1998) compared two doses of dog visits (30 min/ week or 3 × 30 min/week). In both studies, loneliness was assessed using the same validated tool (University of California Los Angeles Loneliness Scale), however, there was insufficient raw data (one study presenting their data graphically) to formally pool the data. Both studies reported significantly less loneliness in those residents who received dog visits compared to residents in the control arms. There was, however, no difference in loneliness scores between the two doses of dog visits, or between the dog or robotic dog visits.

### Morale

Two RCTs reported on the effect of dog visits on morale, both assessed using the Philadelphia Geriatric Centre Morale Scale [65, 72]. The results could not be pooled due to insufficient raw data being presented. Neither study reported any beneficial effect of a weekly dog visit on resident morale.

#### Behavioural

### Agitation

Agitation was assessed in two 12 week parallel RCTs [60, 64], and one crossover RCT (of 2 weeks in each treatment arm) [66]; all three of which investigated the effects of weekly dog visits for residents living with dementia. Agitation was assessed using the Cohen Mansfield Agitation Inventory [60, 66] and the Behavioural Activity Rating Scale [64]. Pooling the data from the parallel trials showed there no evidence that dog visits had an effect on agitation, SMD -0.42 (-1.13 to 0.29;*p* = 0.25; I^2^ = 62%), see Additional File 1: Figure S2. The cluster randomised trial also reported no effect of dog visits on resident agitation.

### Apathy

Two trials evaluated the effect of pet therapy on resident apathy [60, 70]. Both were studies assessing the impact of weekly dog visits over 12 weeks for residents living with dementia. The data could not be pooled due to insufficient reported raw data, however, both studies reported finding no effect of dog therapy on resident apathy.

#### General behaviour

Three studies reported on the effects of pet therapy using composite measures of resident behaviour [66, 70, 72]. All three were investigating the effect of dog visits, and two of the studies had a focus on residents living with dementia [66, 70]. The tools used included a bespoke social behaviour checklist [66], the Nurse Observation Scale for Inpatient Evaluation-30 tool [72], and the Neuro-Psychiatric Inventory [70]. The data was not able to be pooled due to insufficient reported raw data. The findings were mixed. Pope et al.reported significant improvements in overall behaviour in residents after two weeks of dog visits compared to visits with human interaction alone [66]. Valenti-Soler et al. found no significant difference in behaviour after 12 weeks of twice weekly dog visits compared with usual care visits with a therapist alone [70]. Zulauf reported significant improvements in one of the two groups receiving dog visits over 6 weeks, compared to therapist only visits, but the second group receiving dog visits were no different to the therapist only group [72].

#### Interaction/ engagement

Eight studies [54, 60–62, 66, 68, 71, 72] reported outcomes reflecting the effect of pet therapy on resident interaction and engagement, including verbalisation, degree of eye contact, smiling, interaction/touching, and observations of engagement and activity participation. Due to the diversity of measures, and lack of reported pre and/or post raw data, it was not possible to pool the data. The findings were very mixed and there was no consistent evidence of effect. For example, for verbalisation, three studies reported increased aspects of speech during pet therapy compared to control sessions [60, 61, 68] whereas others found no evidence of difference [54, 62, 71]. Similarly, greater levels of eye contact were observed during pet therapy in one study [68], but others found no difference [60, 62].

#### Wellbeing

### Quality of life

Five studies assessed the effect of pet therapy on resident quality of life [58, 59, 64, 69, 70]. Four studies focussed on residents living with dementia. All four involved weekly dog visits, three over a period of 10–12 weeks and for the fourth over a period 9 months [58]. All four used a group-based approach for the intervention. Quality of life was assessed using the QUALID tool [64, 70] and the QOL-AD tool [58, 69]. The fifth study investigated the impact of the resident having either a personal bird or plant to care for, compared to nothing extra, over a period of three months [59]. In this study, the perception of quality of life was assessed using the LEIPAD-II scale. Data from two of the dog studies were able to be pooled [64, 70]. The pooled results show no evidence of effect from the dog visits on quality of life SMD -0.06 (-0.42 to 0.30; *p* = 0.75; I^2^ = 0%), see Additional file 1: Figure S2. The study by Travers et al. of 56 residents found mixed findings on quality of life across its three homes: no effect in one, improved quality of life in another and a reduction in quality of life in the third [69]. In the longer study by Briones et al., both groups improved with time, but there was no difference between those who had received dog-assisted therapy to those who had not [58]. The study by Colombo et al., found that having a bird to look after, resulted in significant improvement across all subscales of the quality of life tool, when compared to looking after a plant or usual care [59].

### Overarching synthesis

**Fig. 2:**
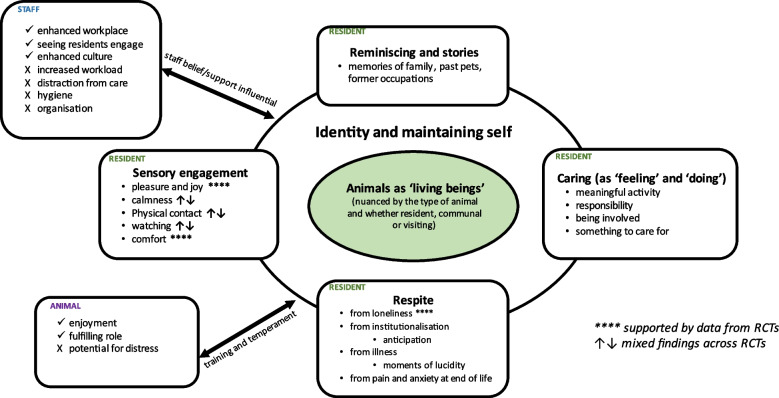
Conceptual model of experiences and meanings of human-animal interactions in care homes

The conceptual model, shown in Fig. 2, presents the synthesised themes identified through the qualitative synthesis and indicates where the quantitative evidence is supportive. Sensory engagement describes the experience that pets and animals had on the senses; with the joy, pleasure and the comfort that the sight and touch of a living animal can bring, along with associated memories that they stir. The pooled evidence from the trial data lends some support to this showing pet therapy can help to reduce anxiety and depression. Two studies also found evidence that pet therapy reduced loneliness. This may be through the sensory engagement that living animals enable but also resonates with the broader concept of respite, in this case respite from loneliness. In addition, one study of residents without dementia reported significant improvement in resident quality of life for those having a canary living with them in their room over three months, compared to being given a plant. This offers support to the theme of ‘respite’; in this case from institutionalisation, as the purpose of the trial was to give the residents something to care for, rather than them ‘being cared for’. This also connects with the theme ‘Caring’ and having ‘something to care for’.

However, as described within some of the qualitative studies, there were instances in which residents did not engage with pets and visiting animals, nor appear to benefit from their presence and this was apparent in some of the trials. The pooled evidence for quality of life showed no overall effect, which stands in contrast to many of the areas within the themes of sensory engagement, respite and caring. In addition, there was no reported benefit from pet therapy on agitation for residents living with dementia in several studies, refuting perhaps the qualitative evidence of animals having a calming effect. There was also no evidence of effect on resident apathy in the trial data, again perhaps at odds with the concept of animals fostering meaningful activity, a strong theme in the qualitative data.

Whilst there were some trials that reported increased engagement and interaction, through speech, eye contact and physical touch, which could potentially lead to or reflect connecting with others and/or maintaining self, there were an equal number of studies that reported no impact. This did not appear to be related to whether the resident was living with dementia or not, nor any specific aspect or length of intervention. Similarly, results assessing the effect of pet or animal visits on various aspects of resident behaviour, were mixed within studies and across studies. There were no RCTS reporting outcomes that could support or refute the experiences of the Staff Perspective theme in the concept model.

## Discussion

This is the first mixed methods systematic review to examine the experiences and effects of pets and animals in older adult residential care settings. A strength of the review is the rich qualitative findings which identified five themes around experiences and meanings of resident-animal interaction in care homes. Engaging with animals had positive effects on the health and wellbeing of residents by providing opportunities for residents to interact with ‘living beings’, to reminisce and share stories, to care for a living creature, have respite, and engage their senses. The conceptual model (see Fig. 2), generated from the process of drawing the qualitative and quantitative evidence together, depicts the resident-animal interaction at the centre, with identity as the global theme that draws the other themes together, and offers an overarching interpretative framework for understanding the health and wellbeing benefits of animal interactions for care home residents. In this section the discussion focusses firstly, on the significance of the relationship between animals and residents; secondly, on how residents maintain identity by living with, and caring for, their pet, reminiscing, participating in meaningful activities with animals, and through the sensory and embodied experiences with the animals; and thirdly, on the importance of care homes adopting a person-centred care approach to resident-animal interactions.

At the core of the conceptual model was the underpinning theme of the animal as a ‘living being’, and the kinship or connectedness that this meant to individual residents. Within human-animal interaction research, there is an implicit assumption that it is ‘something unusual, specific, or even unique about animals’ that brings about change [77]. In our conceptual model, the human-like quality of the animal underpinned the experiences of how pets and animals connected with the residents. Residents’ descriptions of animals as ‘almost human’ may be explained as anthropomorphism which is the “…attribution of human mental states (thoughts, feelings, motivations and beliefs) to non-human animals” ([78], p.437). Although regarded by some as ‘undesirable’, ([79], p.141)Beetz argues that it is the basis for understanding how humans relate to and communicate with animals and build social relationships with them. Residents, and particularly those who were living with their pets in the care homes, spoke about their relationships with their pets in terms of kinship [80], and how they received social and emotional support from their pets. Residents could interpret animal gestures such as a ‘paw on a cheek’ as a sign of emotional support and describe it in similar terms to that of human support. Their evaluations of the animal’s behaviour – often regarded as superior to that of humans – influenced the degree to which residents were attached to their pet. Arguably, relationships with animals were valued because animals were similar to humans and also because they were not [80]. The emotional bonds with their pets were of even greater significance to residents when they felt alone and apart from family and friends, and could even substitute for lost relationships (e.g. with a spouse). Thus, the support and positive social relationships residents experienced with their pets contributed to their wellbeing.

The residents benefitting from this social and emotional support were most likely to be those with a long-term relationship with their own pets and who were living with them in the care home. However, having a relationship with visiting animals was important for some residents too and familiarity could be achieved when the same animals returned on each visit, on a regular schedule over a sustained period of time [40]. Residents indicated that having the animals recognise them enhanced the pleasure they experienced, so regularity and continuity in animal visits is important to ensure that wellbeing benefits are delivered. Interestingly, in these relationships between humans and animals, the animal can be seen as an active participant with its behaviour and responsiveness shaping the relationship with the human [80]. This challenges those studies where the animal has been treated as the “…uniform variable that either is present or absent, as if all [animals] were equivalent, regardless of species, breed, temperament or behaviour” ([78], p.444). According to Serpell et al. (2017), acknowledging the individual characteristics of animals participating in interventions should be an important part of accounting for heterogeneity in RCTs and increasing their methodological rigour [77].

The presence of animals helped residents maintain continuity of self, particularly important as moving to, and living in, a care home can mean that residents experience changes and losses that impacts their identity and wellbeing [81]. For example, residents may experience loss of self-care ability, loss of control or autonomy, loss of meaningful connections [44], and loss of domesticity [46]. In this context, being able to keep their pet in the care home seemed to “…take on increased significance and meaning” for residents ([4], p.9), perhaps by helping residents to maintain a connection between their former life and current one in the care home [44, 82]. However, with many care homes not able to take on animals, some residents were forced to part with their companion animal, which Fox and Ray suggest is akin to the loss of a family member, having a detrimental impact on their health and wellbeing [76]. Residents’ descriptions of their pets as family indicates ‘connectedness and belonging’ and arguably, pets offered emotional support, comfort and security to such an extent that they provided residents with a sense of ‘ontological security’ [80]. That some residents were able to continue caring for their pets and fulfil their caring responsibilities was important, not only for maintaining a degree of autonomy or agency [36] but also for maintaining the identity of ‘pet owner’. Arguably, personal pets are part of the identities of residents in a way that communal and visiting animals could never be and to some extent, resident disinterest in visiting animals may have been recognition that the animal was ‘not theirs’ to which they could link their identity.

However, there is evidence to suggest that visiting and communal animals did enable some residents to reconnect with their former lives through reminiscence and storytelling, and through caring activities. Interestingly, some residents spoke of how important the visiting animals were to them in terms of ‘belonging to something’ [52]. Where residents were involved in the everyday caring routines for communal animals, they were ‘doing’ meaningful activities which may have been a way of linking with former routines and replacing the loss of other responsibilities [81]. For some, such activities could be described as ‘identity work’ [83], and meaningful activities have been identified as supporting personhood for residents with dementia by enabling them “to continue to be who they are” ([84], p.12). In some cases, there was an assumption that for residents to find interacting with the animal meaningful, they had to have experience of animals in the past, but this was not always the case [39, 48], which fits with Strick et al.’s assertion that ‘meaningful occupation’ can also support an ‘evolving and changing identity’ [85].

The importance of touch in resident encounters with animals, and the pleasure and comfort derived from touch, particularly for those residents with dementia, highlights how it can enhance the wellbeing of care home residents. In care settings, residents can experience ‘touch deprivation’ [86], in that they are more likely to receive instrumental, task-oriented touch associated with the routine tasks of caring, than caring touch for ‘reassurance or comfort’ ([87], p.543). Touch in the context of carers providing daily assistance with washing, dressing, eating and walking has been described as ‘bodywork’ [88]. That residents enjoyed the animals’ ‘snuggles’ and ‘cuddles’ suggest that such physical interactions restored an ‘element of touch’ to their lives ([46], p.115), and may even have acted as a “substitute for the lack of affectionate human touch” ([89], p.10). For some residents with dementia, cuddling and petting the animals enabled them to connect to the present and be aware of the animal and its body. In that moment, residents could engage in conversation, show awareness of, and respond to, the situation and express care for the animal through an “embodied knowledge of how to take care” ([90], p.329). This resonates with the concept of embodied selfhood [91] and how people with dementia use non-verbal behaviour to engage with, and connect to, the world.

The physical contact with an animal could also enable residents to connect with ‘life past’ and evoke ‘dormant’ memories and feelings which could bring sadness as well as joy [47]. Swall et al. draws on embodiment to explain how physical contact with animals could stimulate memory:

“To feel, see and hear the dog reveals feelings and expressions from a ‘whole’ human being, allowing them to connect with…memories earlier in life when they were younger and before they contracted AD [Alzheimer’s Disease]. Their existence and awareness might be due to the sense of being a ‘whole’ human being with the dog felt through their senses, which in turn connects with inner feelings and memories that they reveal when the dog is close”. ([47], p.89).

Downs argues that embodiment is important for understanding ‘continuity of self’ in dementia and the embodied nature of the human-animal interaction enabled residents living with dementia to express something of their identity [92]. There is increasing recognition that the body and embodiment are central to living with dementia with implication for care practices which, according to Downs ([92], p.368), should be “…person-centred in the sense of affirming personhood and sense of self.” Providing opportunities for residents with dementia to interact with animals is potentially a person-centred approach to strengthen a sense of self-identity [49].

A person-centred approach to care is about staff recognising each resident in the care home as a unique individual with likes and dislikes, understanding his/her life story, and appreciating the resident’s preferences in order to offer activities that match his/her interests and capacities [93, 94]. The overwhelming view of the staff in Fossey & Lawrence’s ([37], p.250) study was that involving animals should be “…facilitated to ensure resident’s’ choices and preferences were respected,” yet the evidence suggests that in practice, the attitudes of staff towards animals were likely to determine whether they were considered as part of person-centred care. Additionally, the willingness of care home managers to support resident pets or visiting animals was a critical factor. These findings are supported by Buist et al.’s research on the implementation of green care farm characteristics in long-term care settings which found that staff flexibility and ability to deliver person-centred care, management commitment and vision, and flexible approaches to risk and safety facilitated change and innovation [95]. Notably, Fossey & Lawrence ([37], p.322) observed that staff often presented health and safety concerns as “a default response that negated the need to consider the topic further.” The extent to which health and safety risks were experienced as a perceived or actual barrier is also likely to be closely related to a care home’s approach to person-centred care. According to Ettelt et al.’s typology of care home approaches, in the task-oriented, risk averse care homes, person-centred care happened at the ‘periphery’, with managers highlighting the tension between person-centred care and risk management [96]. For Fox & Ray ([76], p.212), risk reduction is paramount when residents are viewed as a “homogenous vulnerable group requiring protection from harm” and, in the words of Freedman et al. ([38], p.1976), that is because “risk reduction is valued above identity.”

Person-centred care should be an important influence when developing a policy for resident and visiting animals [97]. Given that the evidence suggests that interacting with animals is more than simply an activity that residents do and is important for ‘who they are’ [38], care homes should think carefully about the animal interactions that best match individual residents’ needs and respect individual choice and autonomy. Arguably, the presence of resident pets and communal animals offered residents the possibility of spontaneous interactions on their own terms, whereas interactions with visiting animals were more likely to be structured and time-bounded. While these visits were a highlight for some, they were, for others, ‘another activity’ that had been organised *for them* by management [52]. This reinforces how a sense of agency was important for some residents and where possible care homes should aim to facilitate participation in animal visits as a ‘meaningful choice’ for residents. Visits from therapy animals for residents with dementia tended to be structured, as in one study where dog visits were prescribed with each referral made by a nurse, and individualised for each resident with a specific purpose such as increase alertness or decrease anxiety [47–49]. The dog handlers indicated that by knowing the residents they aimed to tailor each visit to the individual and respond to the residents’ emotions and feelings. The importance of ‘learning to read the residents’ signals’ was observed by one dog handler [39], and demonstrated that the dog handlers sought to support residents with dementia to exercise agency [98]. The challenge for care home staff is how best to balance supporting residents with dementia to exercise agency and motivate them to engage with animal therapy [85].

Previous systematic reviews of animals in the residential care setting have identified the promise of animal-assisted interventions but have largely focussed on quantifiable outcomes and have focussed on specific populations. Whilst most highlight the lack of rigorous studies, the reviews suggest there is weak evidence that targeted animal -assisted therapies might be effective for older adults with dementia in improving social functioning [15, 18, 19], depression [20, 23] and agitation [16, 20, 99]. Borgi et al. (2018) reviewed the evidence of dog-assisted interventions on older adults with depression, including those in care homes [21]. Despite considerable heterogeneity amongst the studies, the authors concluded that dog assisted interventions of at least four weeks or more, had beneficial effects on depressive symptoms for those living in the community as well as those in residential care, and for those with cognitive impairment of any level, in line with findings from previous reviews [23, 100], and further supported by Jain et al. (2019) [22]. The review by Jain et al., included four qualitative studies, from which the authors drew three themes: dogs visiting the home served as ‘transitional objects’ supplementing otherwise missing interaction, dogs were ‘therapeutic’ in creating a good moment and reducing stress, and lastly the importance of the care home environment in facilitating the most out of dog-assisted interventions [22]. However, there is a recognised dearth of qualitative reviews on the experience of pets and animals in this setting [22, 101]. It seems intuitive that we need to understand the value of pets and animals to residents in long-term care before we try to assess if they are ‘effective’. The qualitative synthesis and conceptual model from our review brings new insight into this area.

## Strengths and limitations of review

A strength of this review is that it followed best practice guidelines for both quantitative and qualitative syntheses and was informed by stakeholders. We searched widely for relevant literature and did not limit by date or language. It is also the first systematic review to bring together qualitative and qualitative evidence on human-animal interactions in the long-term care setting: being inclusive of all type of animal interactions, from resident animals and pets through to structured therapeutic visits. It highlights the value of qualitative research in capturing the richness, diversity and similarities in resident experiences of animal interactions. Six of the included papers were drawn from two studies [47–52] and Table 2 shows that these papers made similar contributions to the thematic synthesis, apart from one [50].

The potential for animals to elicit negative responses from residents received little attention in the studies. The benefits of animals for residents in long-term care has “almost universal acceptance” ([102], p.151) with the public eager to consume the ‘feel-good’ stories published by the mass media [103]. However, this may present researchers with a ‘double-edged sword’ ([77], p.7), in that a ready audience for their findings may exert a subtle pressure to report positive findings and overlook the”…need to document what might seem the obvious” ([102], p.151). Whilst there were examples of residents becoming over attached or possessive with the animals, and others simply disinterested, the focus was more on the potential benefits. Future research should address this need to document some of the resident-specific challenges of introducing animals into long-term care settings.

The potential discrepancies observed across the qualitative and quantitative syntheses may in part be due to the appropriateness and sensitivity of the outcome measures being used in the trials which may not reflect the value or impact that are important to the participants. They also may be a reflection that the sample sizes in the trial evidence were too small to detect meaningful differences.

There also is a possibility that the difference is explained by the wide and diverse nature of the human-animal interactions that have been explored and assessed across studies to date. The type of animal, the nature of the interaction (individual vs communal, therapy vs activity), and the personality and history of the resident, inevitably leads to a heterogeneity of experience and impact which is difficult to capture and unlikely to be uniform. This is acknowledged, to some extent, in the conceptual model (Fig. 2) which proposes that the experiences and impacts of the human-animal interaction are nuanced by the type of animal interaction which may vary and change across time. Arguably, more research attention should focus on understanding the strengths and limitations of the different types of human-animal interactions themselves.

There was little in the studies about how living in, or visiting, care homes impacted the health and wellbeing of the animals. There is a paucity of research on the possible ill-effects of human interaction on animals [103] and the dominant utilitarian approach in research focuses on ‘what can animals do for us?’ ([75], p.39). Staff raised the welfare of resident pets and the potential for neglect [37] but did not discuss any other aspects of animal wellbeing. Gorman ([104], p.318) counsels against assuming that animals do not receive any benefits from their encounters with humans in a care setting, by arguing that it “…gives rise to the view of animals as passive and lacking agency, simply receiving human action.” He believes that human-animal relations have the potential to be ‘reciprocally beneficial’. The potential for animals being active partners within the relationship and receiving benefit is likely to vary according to the type of animal and its role in the care setting and in this review, some animals were pets belonging to individuals living in the care homes, others were communal animals and part of the care home environment, and others were visiting with either volunteers or trained animal handlers. Arguably, the chickens in the HENPOWER project were less likely to have a mutually beneficial relationship with residents in that they were part of the homes’ outdoor spaces and residents were less likely to interact with the chickens themselves [104]. Understanding more about the experience of animals is essential to ensure that they too have opportunities to mutually benefit from interacting with care home residents. There was also very little in the studies that considered the perceptions and experiences of family members, and only a few studies examined the experiences and perspectives of the volunteers [46] and the dog handlers [39, 48, 50].

The review is limited by the quality of the included quantitative studies. In particular, many of the randomised trials were small, and of short duration, with little or no follow-up. In addition, for the majority of the trials both the residents and researchers were aware of group allocation. Another limitation of the quantitative evidence is the appropriateness of outcome measures, which may not reflect the value or impact that are important to the participants. The qualitative research included in the review was generally of higher quality.

## Conclusion

This systematic review demonstrates that animals can significantly impact the health and wellbeing of some care home residents. Residents had meaningful relationships with pets, resident and visiting animals, which were experienced as embodied and emotionally supportive, and from which they derived pleasure and comfort. Interacting with animals offered residents a way to maintain a sense of self in the care homes, and with support, residents with dementia could also express their identities. Facilitating residents to interact with animals as part of person-centred care may also help residents to feel ‘at home’ in the care home, which, according to Cooney, [93] is associated with ‘continuity’, ‘preserving personal identity’, ‘belonging’ and ‘being active and working.’ How care homes facilitate the human-animal interaction, whether it is pets living in the home with their owners or communal animals, or animals as visitors—designated as ‘activity’ or ‘therapy’, the critical factor is that residents’ agency is recognised and respected. Where animals are not a viable option for care homes, robotic animals that mimic ‘living’ animals and respond to human touch, might be a viable alternative [33]. Any care homes considering resident or visiting pets and animals should have an animal policy in place, as recommended by the UK animal charity Blue Cross [105], and establish connections with local vet services. In conclusion, care homes should consider carefully how to encourage human-animal interactions as it will impact the daily functioning and wellbeing of those living, working and visiting long-term care environments.

## Supplementary Information

**Additional file1: FileS1.** Search strategy for MEDLINE. **Table S1.** Search Summary. **Table S2.** ENTREQ Statement (Enhancing the transparency in reporting the synthesis of qualitative research). **Table S3.** Quality Appraisal of Included Qualitative Studies. **Table S4.** Quality Appraisal of Randomised Trials. **Table S5.** Illustrative Quotations from Primary Studies according to Analytical Themes. **Figure S1.** Thematic Network. **Figure S2.** Meta-analyses showing effects of animal assisted intervention on outcomes of depression, anxiety, agitation and quality of life. **File S2.** PRISMA checklist.

## Data Availability

This file has five tables referenced in the main paper and shows the search summary, the ENTREQ statement, the quality appraisal of the qualitative studies using the Wallace criteria, the Risk of Bias, and illustrative quotations from the primary studies according to analytical themes. This file has two figures referenced in the main paper and shows the thematic network and the metanalyses showing effects of animal assisted intervention on outcomes of depression, anxiety, agitation and quality of life. There are two files, one for the search strategy for MEDLINE and one for PRISMA.
