# Supplementary material for: What are the effects of animals on the health and wellbeing of residents in care homes? A systematic review of the qualitative and quantitative evidence
Source: BMC Geriatr. 2023 Mar 25;23:170. doi: 10.1186/s12877-023-03834-0 (PMC10038779; doi:10.1186/s12877-023-03834-0)
Supplement: Supplementary file 1 — Additional file1: FileS1. Search strategy for MEDLINE. Table S1. Search Summary. Table S2. ENTREQ Statement (Enhancing the transparency in reporting the synthesis of qualitative research). Table S3. Quality Appraisal of Included Qualitative Studies. Table S4. Quality Appraisal of Randomised Trials. Table S5. Illustrative Quotations from Primary Studies according to Analytical Themes. Figure S1. Thematic Network. Figure S2. Meta-analyses showing effects of animal assisted intervention on outcomes of depression, anxiety, agitation and quality of life. File S2. PRISMA checklist. [file 12877_2023_3834_MOESM1_ESM.docx]

**Additional File 1**

File S1: Search strategy for MEDLINE

Table S1: Search Summary

Table S2:  ENTREQ Statement (Enhancing the transparency in reporting the synthesis of qualitative research)

Table S3: Quality Appraisal of Included Qualitative Studies

Table S4: Quality Appraisal of Randomised Trials

Table S5: Illustrative Quotations from Primary Studies according to Analytical Themes

Figure S1: Thematic Network

Figure S2: Meta-analyses showing effects of animal assisted intervention on outcomes of depression, anxiety, agitation and quality of life

File S2: PRISMA checklist

**File S1: Search strategy for MEDLINE**

Database: Ovid MEDLINE(R) Epub Ahead of Print, In-Process & Other Non-Indexed Citations, Ovid MEDLINE(R) Daily and Ovid MEDLINE(R) <1946 to Present>

Search Strategy:

--------------------------------------------------------------------------------

1 (Animal* or pet*).tw. (1101436)

2 (cat or cats).tw. (129838)

3 (dog or dogs).tw. (196858)

4 canine*.tw. (78367)

5 bird*.tw. (64955)

6 robo*.tw. (30516)

7 exp Cats/ (132552)

8 exp Dogs/ (314801)

9 exp Fishes/ (162461)

10 exp Bonding, Human-Pet/ (1683)

11 (fish or fishes).tw. (145492)

12 exp Animal Assisted Therapy/ (354)

13 1 or 2 or 3 or 4 or 5 or 6 or 7 or 8 or 9 or 10 or 11 or 12 (1820437)

14 ((Nursing or geriatric*) adj2 home*).tw. (28784)

15 ((Nursing or geriatric*) adj2 unit*).tw. (3572)

16 ((Nursing or geriatric*) adj2 facilit*).tw. (4061)

17 ((older or senior* or elder* or dement* or Alzheimer*) adj2 institut*).tw. (3111)

18 ((older or senior* or elder* or dement* or Alzheimer*) adj2 resident*).tw. (5396)

19 ((older or senior* or elder* or dement* or Alzheimer*) adj2 unit*).tw. (974)

20 ((older or senior* or elder* or dement* or Alzheimer*) adj2 facilit*).tw. (722)

21 ((older or senior* or elder* or dement* or Alzheimer*) adj2 home*).tw. (3898)

22 14 or 15 or 16 or 17 or 18 or 19 or 20 or 21 (44442)

23 (Care adj2 home*).tw. (24821)

24 ((Long-term or resident*) adj2 care).tw. (24797)

25 (Institut* adj2 (care* or resident* or unit* or facilit* or home*)).tw. (11402)

26 23 or 24 or 25 (56873)

27 (Old* adj2 (people* or person* or adult*or resident*)).tw. (37505)

28 (elder* adj2 (people* or person* or adult*or resident*)).tw. (25368)

29 (geriatric* adj2 (people* or person* or adult*or resident*)).tw. (187)

30 (senior* adj2 (people* or person* or adult*or resident*)).tw. (256)

31 27 or 28 or 29 or 30 (60907)

32 exp Aged/ (2744030)

33 31 or 32 (2759714)

34 26 and 33 (23114)

35 exp Home Nursing/ (9385)

36 exp Homes for the Aged/ (12645)

37 22 or 34 or 35 or 36 (73955)

38 13 and 37 (612)

***************************

<1>

Unique Identifier

28433400

Record Owner

From MEDLINE, a database of the U.S. National Library of Medicine.

Status

Publisher

Authors

Rantanen P; Parkkari T; Leikola S; Airaksinen M; Lyles A.

Authors Full Name

Rantanen, Pekka; Parkkari, Timo; Leikola, Saija; Airaksinen, Marja; Lyles, Alan.

Institution

Rantanen, Pekka. Validia Rehabilitation, Helsinki, Finland.

Parkkari, Timo. Clinius Ltd., Helsinki, Finland.

Leikola, Saija. Pharmac Finland Oy, Vantaa, Finland.

Airaksinen, Marja. Clinical Pharmacy Group, Division of Pharmacology and Pharmacotherapy, Faculty of Pharmacy, University of Helsinki, Helsinki, Finland.

Lyles, Alan. School of Health and Human Services, College of Public Affairs, University of Baltimore; Docent, University of Helsinki, Helsinki, Finland. Electronic address: calyles@ubalt.edu.

Title

An In-Home Advanced Robotic System to Manage Elderly Home-Care Patients' Medications: A Pilot Safety and Usability Study.

Source

Clinical Therapeutics. , 2017 Apr 19.

Abbreviated Source

Clin Ther. , 2017 Apr 19.

NLM Journal Name

Clinical therapeutics

Publishing Model

Journal available in: Print-Electronic

Citation processed from: Internet

NLM Journal Code

cpe, 7706726

ISO Journal Abbreviation

Clin Ther

Country of Publication

United States

Keyword Heading

aged

home-care services

medication adherence

medication therapy management

robotic system

Abstract

PURPOSE: We examined the safety profile and usability of an integrated advanced robotic device and telecare system to promote medication adherence for elderly home-care patients.

METHODS: There were two phases. Phase I aimed to verify under controlled conditions in a single nursing home (n = 17 patients) that no robotic malfunctions would hinder the device's safe use. Phase II involved home-care patients from 3 sites (n = 27) who were on long-term medication. On-time dispensing and missed doses were recorded by the robotic system. Patients' and nurses' experiences were assessed with structured interviews.

FINDINGS: The 17 nursing home patients had 457 total days using the device (Phase I; mean, 26.9 per patient). On-time sachet retrieval occurred with 97.7% of the alerts, and no medication doses were missed. At baseline, Phase II home-dwelling patients reported difficulty remembering to take their medicines (23%), and 18% missed at least 2 doses per week. Most Phase II patients (78%) lived alone. The device delivered and patients retrieved medicine sachets for 99% of the alerts. All patients and 96% of nurses reported the device was easy to use.

IMPLICATIONS: This trial demonstrated the safety profile and usability of an in-home advanced robotic device and telecare system and its acceptability to patients and nurses. It supports individualized patient dosing schedules, patient-provider communications, and on-time, in-home medication delivery to promote adherence. Real time dose-by-dose monitoring and communication with providers if a dose is missed provide oversight generally not seen in home care.

Copyright © 2017 The Authors. Published by Elsevier Inc. All rights reserved.

ISSN Electronic

1879-114X

ISSN Linking

0149-2918

Publisher Item Identifier

S0149-2918(17)30202-3

Digital Object Identifier

https://dx.doi.org/10.1016/j.clinthera.2017.03.020

Publication Type

Journal Article.

Article Identifier

S0149-2918(17)30202-3 [pii]

10.1016/j.clinthera.2017.03.020 [doi]

Publication Status

aheadofprint

Publication History Status

2016/12/16 [received]

2017/03/17 [revised]

2017/03/22 [accepted]

Language

English

Electronic Date of Publication

20170419

Date of Publication

2017 Apr 19

Date Created

20170423

Entrez Date

2017/04/24 06:00

MeSH Date

2017/04/24 06:00

Create Date

2017/04/24 06:00

Year of Publication

2017

Revision Date

20170423

Update Date

20170425

Link to the Ovid Full Text or citation:

Click here for full text options

Link to the External Link Resolver:

WebBridge

**Table S1. Search Summary**

(Bethel AC, Rogers M, Abbott R. Use of a search summary table to improve systematic review search methods, results, and efficiency. J Med Libr Assoc. 2021 Jan 1;109(1):97-106. doi: 10.5195/jmla.2021.809. PMID: 33424470; PMCID: PMC7772975)

**Table S2. ENTREQ Statement (Enhancing the transparency in reporting the synthesis of qualitative research)**

| **Item** | **Guide and description** | **Page no** |
| --- | --- | --- |
| **1. Aim** | To synthesise the qualitative and quantitative research evidence on the effects of animals on the health and wellbeing of care home residents. | 3 |
| **2. Synthesis methodology** | Thematic synthesis was used to synthesise the qualitative studies. | 4 |
| **3. Approach to searching** | Pre-planned, comprehensive search strategy to seek all available studies in the published literature according to a pre-planned, online PROSPERO protocol (CRD42017058201). | 3 |
| **4. Inclusion criteria** | Phenomenon of interest: Views, experiences and perceptions of interacting with animals of older people in care homes, their families and carers, and care home staff.  Population: Older people resident in care homes, their families and carers, and care home staff.  Language: English language only.  Year: No exclusion based on year of publication.  Types of studies: Qualitative studies using recognised methods of qualitative data collection and data analysis. | 4 |
| **5. Data sources** | Electronic databases: MEDLINE, EMBASE, PsycINFO, SPP (via OvidSP), CINAHL, AgeLine (via EBSCOhost), CDSR, CENTRAL, DARE (via Wiley Online, Cochrane Library), ASSIA (ProQuest), Web of Science Core Collection (SCI-Expanded, SSCI, A&HI, CPSI-S, CPSI-SSH, ESCI), SCOPUS and ProQuest Dissertations and Thesis Global.  Supplementary methods: forward and backward citation chasing of each included article was performed.  Last search July 2020.  An exhaustive search of the literature was completed. | 3 |
| **6. Electronic search strategy** | Search strategy is described in detail in Additional File 1: File S1 & Table S1. | File S1  Table S1 |
| **7. Study screening methods** | Four reviewers (RA, SP, NO and RW) independently screened titles and abstracts against eligibility criteria. The full text of articles initially considered as meeting the inclusion criteria were retrieved and the eligibility criteria applied in the same way. Discrepancies at both stages were discussed and resolved with another reviewer (JTC) where necessary. | 4 |
| **8. Study characteristics** | Details of the study characteristics are provided in Table 1. | 33 |
| **9. Study selection results** | Figure 1 outlines the study selection process in a PRISMA* flow diagram. | 46 |
| **10. Rationale for appraisal** | The purpose of the quality appraisal was to critically appraise the qualitative studies. | 4 |
| **11. Appraisal items** | The Wallace criteria was used to critically appraise the qualitative studies. | 4 |
| **12. Appraisal process** | The quality appraisal was conducted independently by two reviewers (RA & NO) and consensus reached by discussion. | 4 |
| **13. Appraisal results** | The quality appraisal results are available in Additional File: Table S3. We did not exclude any articles on the basis of quality as we believed that all studies may contribute some important insights to the phenomenon of interest. | Table S3 |
| **14. Data extraction** | All content in the results and discussion sections of the included papers were considered as data for analysis. These data were extracted into a bespoke data extraction form. Data extraction was completed by one reviewer and checked by another.  Information extracted: date of publication, country of conduct, study aim, sample (size and characteristics), intervention (duration and type), dementia focus, study design (methods and analysis), were extracted from the included studies and are presented in Table 1. | 4 |
| **15. Software** | Not used. |  |
| **16. Number of reviewers** | Two reviewers (RA & NO) read all the included studies in detail; one reviewer extracted data from the papers which was then checked by the other reviewer, both meeting to discuss the findings, and reach a consensus. | 4 |
| **17. Coding** | Line-by-line coding was carried out as the first step in the thematic synthesis process. | 4 |
| **18. Study comparison** | The data were compared across studies and two reviewers (RA, NO) discussed the similarities, differences and connections. A thematic network approach was used to structure and depict the themes (Additional File 1: Figure S1). The reviewers brought the qualitative and quantitative findings together in an overarching synthesis and using the qualitative findings as a framework, created a conceptual model (Figure 2) which showed where the quantitative data supported or refuted the qualitative findings. | 4-5 |
| **19. Derivation of themes** | The process of developing the themes was inductive: groups of related codes were combined into descriptive themes and these were then re-interpreted inductively to develop analytical themes. We used a thematic network approach as an aid to discussion and an illustrative tool. We arranged the descriptive themes into networks, grouped around the analytical themes, and then grouped these around a ‘global’ or ‘macro’ theme. The global theme was the theme that interpreted the other themes (Additional File 1: Figure S1). | 4-5 |
| **20. Quotations** | Direct quotes from the participants – residents, care staff, animal handlers and volunteers - are presented in the Results section of the manuscript and in more detail in Additional File: Table S5. | Table S5 |
| **21. Synthesis outputs** | The qualitative evidence and quantitative evidence were brought together in an overarching synthesis. There is some overlap between the qualitative and quantitative evidence bases. | 18-19 |

*PRISMA – Preferred Reported Items for Systematic Reviews and Meta-Analyses.

**Table S3 Quality Appraisal of Included Studies**

| **Author(s)** | **Is the research question clear?** | **Theoretical perspective of author clear?** | **Theoretical perspective influenced the study design?** | **Is the study design appropriate?** | **Is the context adequately described?** | **Sample adequate to explore range of subjects/settings?** | **Sample drawn from appropriate population?** | **Data collection adequately described?** | **Data collection rigorously conducted?** | **Data analysis rigorously conducted?** | **Findings substantiated/ limitations considered?** | **Claims to generalizability follow from data?** | **Ethical**  **issues**  **addresse*d*?** |
| --- | --- | --- | --- | --- | --- | --- | --- | --- | --- | --- | --- | --- | --- |
| Casey et al (2018) | Y | N | CT | Y | Y | Y | Y | Y | Y | CT | Y  Y | Y | Y |
| Cook et al (2013) | Y | Y | Y | Y | Y | Y | Y | Y | Y | Y | Y  N | Y | Y |
| Dookie (2013) | Y | Y | Y | Y | Y | Y | Y | Y | Y | Y | Y  Y | Y | Y |
| Fossey & Lawrence (2013) | Y | N | N | Y | Y | Y | Y | Y | Y | Y | Y  Y | Y | N |
| Freedman et al (2021) | Y | Y | Y | Y | Y | Y | Y | Y | Y | Y | Y  Y | Y | Y |
| Gundersen & Jonhannessen (2018) | Y | N | CT | Y | Y | Y | Y | Y | Y | Y | Y  Y | Y | Y |
| Kawamura (2009) | Y | Y | Y | Y | Y | N | Y | Y | Y | Y | CT  Y | Y | Y |
| Kendzioski (1999) | Y | Y | Y | Y | Y | N | Y | Y | CT | Y | N  Y | Y | Y |
| Kongable et al (1990) | Y | N | N | Y | Y | N | Y | Y | CT | CT | N  Y | CT | N |
| Pitheckoff et al (2018) | Y | N | N | Y | Y | N | Y | Y | Y | Y | Y  Y | Y | Y |
| Pooley (2007) | Y | Y | Y | Y | Y | CT | Y | Y | Y | CT | CT  Y | Y | N |
| Roenke & Mulligan (1998) | Y | N | N | Y | Y | N | Y | Y | CT | Y | Y  Y | Y | Y |
| Savishinsky (1985) | Y | N | N | Y | Y | CT | Y | Y | Y | CT | Y  N | Y | N |
| Swall et al (2015) | Y | Y | Y | Y | Y | Y | Y | Y | Y | Y | Y  Y | Y | Y |
| Swall et al (2016) | Y | Y | Y | Y | Y | Y | Y | Y | Y | Y | Y  Y | Y  Y | Y |
| Swall et al (2017) | Y | Y | Y | Y | Y | Y | Y | Y | Y | Y | Y  Y | Y | Y |
| Swall et al (2019) | Y | CT | CT | Y | Y | Y | Y | Y | Y | Y | Y  Y | Y | Y |
| Wong (2015) | Y | Y | Y | Y | Y | Y | Y | Y | Y | Y | Y  Y | Y  Y | Y |
| Wong & Breheny (2021) | Y | Y | Y | Y | Y | Y | Y | Y | Y | Y | Y  Y | Y | Y |
| Zando (2017) | Y | N | N | Y | Y | N | Y | Y | CT | CT | CT  Y | Y | N |

Y –Yes; N – No; CT – Cannot tell

**Table S4: Risk of Bias for included quantitative studies**

|  | Cochrane risk of bias | | | | | | Other bias | |
| --- | --- | --- | --- | --- | --- | --- | --- | --- |
|  | **Random sequence generation** | **Adequacy of allocation concealment** | **Blinding of participants and personnel** | **Blinding of outcome assessment** | **Incomplete outcome data** | **Selective reporting** | **Baseline characteristics similar (or differences accounted for)** | **Intention to treat analysis used or missing data handled appropriately** |
| Andrysko 1982 | Low | ? | High | High | High | Low | ? | No |
| Banks 1998 and 2002 | Low | ? | High | High | Low | Low | Yes | No |
| Banks 2008 | ? | ? | High | High | Low | Low | ? | Yes |
| Bumsted 1998 | Low | ? | High | High | ? | Low | ? | No |
| Briones 2018 | Low | ? | High | High | Low | Low | Yes | Yes |
| Colombo 2006 | ? | ? | High | ? | Low | Low | Yes | ? |
| Friedman 2015 | Low | ? | High | High | Low | Low | Yes | Yes |
| Greer 2002 | ? | ? | High | High | Low | Low | NA | ? |
| Johnson 1997 | ? | ? | High | High | High | Low | Yes | ? |
| Le Roux 2009 | ? | ? | High | High | Low | Low | ? | ? |
| Olsen 2016 | Low | Low | High | High | Low | Low | Yes | Yes |
| Panzer-Kaplow 2000 | ? | ? | High | High | Low | Low | Yes | ? |
| Pope 2016 | ? | ? | High | High | Low | Low | Yes | Yes |
| Thodberg 2015a & b | Low | Low | High | High | Low | ? | Yes | No |
| Travers 2013 | Low | ? | High | High | Low | Low | Yes | Yes |
| Valenti-Soler 2015 | Low | Low | High | Low | Low | High | Yes | ? |
| Wall 1994 | ? | ? | High | High | Low | Low | Yes | Yes |
| Zulauf 1987 | ? | ? | High | High | ? | Low | ? | ? |

KEY Low – low risk of bias; ? – unclear risk of bias; High – High risk of bias; NA not appropriate

**Table S5. Illustrative Quotations from Primary Studies according to Analytical Themes**

| Analytical Theme/Descriptive theme | Data |
| --- | --- |
| 1. Animals as ‘living beings’ | We lived in green fields and she grew up in freedom. But she’s an old lady like me now. You grow old in the environment you live in, and the cat does the same, *they’re no different to human beings*. (Margaret, Resident) (Freedman et al, 2021: 1968 ) (reviewer emphasis)  When I’ve been down in the dumps, *she knows* because she comes and puts her paw on my cheeks and she’s sorry for me. (Margaret, Resident) (Freedman et al, 2021: 1973) (reviewer emphasis)  Right after my mom died I needed something. So I used the rabbit to heal me and it did. I held the rabbit and we prayed and we talked and talked and I talked to the rabbit. (Ms A, Resident) (Pitheckoff et al, 2018: 1570)  “You can make ah acquaintances with ‘em, *make friends*.” (Ms. D, Resident) She felt she could bond and communicate with the rabbits because they appeared to be interested in her. (Pitheckoff et al, 2018: 1570)  What a cute kitty! It’s purring. He’s happy…He has such wonderful eyes, so beautiful. They seem to me *almost like a person*. They’re almost human. There’s nothing like an animal....They have a *sense of knowing* whether you like them or not…They have personality…It’s amazing to me how they make you understand them…and they know you. They’re pretty intelligent, that’s if you treat them right. (Jane, Resident) (Roenke & Mulligan, 1998: 35) (reviewer emphasis)  *He’s smart that dog*…When he comes, he’s better than the kids…You tell him to sit and he sits…(Karen, Resident) (Roenke & Mulligan, 1998: 36) (reviewer emphasis)  For many residents, the animals answer a need that photographs and flowers cannot satisfy, a desire, as one woman expressed it, for ‘*something living that I can name’*. (Savishinky, 1985: 124-5) (reviewer emphasis)  Many residents…speak of their animals as sources of moral value: they are praised for giving and eliciting love, for demonstrating loyalty and trust, for teaching people how to care and be kind, and for offering opportunities to engage in life in a positive way. (Savishinsky, 1985: 120-1)  Escaping from everyday life, finding a friend in the dog and talking about thoughts and experiences of the hard everyday life on the ward. Escape to a meaningful and personal reality with a friend that one does not wish to share with anyone, and appreciating and *preferring the dog to human beings*. (Swall et al, 2015: 88) (reviewer emphasis) |
| 2. Reminiscence and |  |
| storytelling | The chickens were in a big box. Me brother brought them in from the village. And he got on the bus and all these little chicks were going ‘cheep, cheep, cheep.’ And me brother had to put his hand over the top of the box and he got off the bus, he was glad to get off the bus and he got home to me mother’s and put them beside the fireplace, near the fire and it was lovely and warm for them. Me sister, who was young, banged the box and they were running all around the fireplace, and all around the living room, so me mum put her pinny on and she was collecting them up into her pinny pocket. (Resident) (Cook et al, 2013: 56).  You used to wring their [chickens] necks and pluck them, well I had brothers and we used to pluck them and then I used to cook them for Sunday dinner. (Resident) (Cook et al, 2013: 56)  And so when I got this one I thought if I call him Ben, which is the name of the last one, who died, it would help remind me of my dog and my late wife you see because she picked the name. (Jack, Resident) (Freedman et al, 2021:1967)  This is my fourth cat. Because my twin wanted a cat, goes out with the girls and it was left to me and Dad. I said: ‘You wanted a cat, you’ve got to look after it.’ He said: You do it [Daniel], you’re better at that stuff than me.’ Ever since then I’ve been the cat guy. (Daniel, Resident) (Freedman et al, 2021: 1967)  I wouldn’t have gone anywhere without her. We came together, it’s as simple as that. (Molly, Resident) (Freedman et al, 2021: 1968)  One of the female residents suffers from severe dementia, but sitting with the dog, *she starts talking about the dog she used to have at home.* Normally she doesn’t talk much, but then she talks without break. It is incredible. (Nurse) (Gundersen & Johannessen, 2018: 107) (reviewer emphasis)  [Someone] gave us white bunnies for the kids. I don’t know how they got loose out of their box so they went all over the yard. They were real pretty. (Mrs H, Resident) (Pitheckoff et al, 2018: 1571)  We’d put him someplace and we’d say ‘stay there now.’ Then we’d go and hide, and then we’d say ‘okay come out now.’ He would go along and find us…It was so funny when I kneeled down to say my prayers, and he’d come and he’d sit right there and he’d wait until I was done…We’d go into my bed, and I had a place for him on the floor next to the bed. He’d go right to the back of the bed and lay down on the floor. You know you love them just like your children (Alice, Resident) (Roenke & Mulligan, 1998: 37-8)  I can remember when I milked the cows. It made me feel good. Especially on a warm day. (Resident) (Savishinky, 1985: 117).  That’s very kind, but I’d rather that you didn’t. Animals are too sad for me. I mean they remind me too much of the ones I had. It’s painful. I’d rather not if you please. (Resident) (Savishinky, 1985: 122) |
| 3. Caring | Resident pets |
| Responsibility | …it obviously satisfies the need to take care of something that needs me…I’ve noticed that it’s not just something that I enjoy, but I’ve learned that it makes me feel good to be helping something other than myself…To be needed, and to be helpful, and you know I find that I realized um…that without them I’m missing that feeling of looking after something. (Resident) (Dookie, 2013: 198)  …participants claimed that only they understood how to feed or walk their pet. As a result, participants seemed to feel that their pets were reliant on them and that they therefore had a duty of care. (Freedman et al, 2021: 1970)  You still feel responsibility towards your own animal and a strange sense of possession even though you have to share him with everyone else…especially if we all live in one complex. (Diana, Resident) (Freedman et al, 2021: 1971)  …We are happy for everybody to share him because when we die, we know he’s still going to be loved and looked after. (Diana, Resident) (Freedman et al, 2021: 1972)  I give her [Jasper, the resident dog] a treat, talk to her, pet her, tell her she’s a good dog, and thank her for visiting me, like *she really belongs to me*. I guess everyone pretty much likes him [sic]; even the ones that don’t feed him – they like him. (Stella) (Kendziorski, 1999: 35) (reviewer emphasis)  [a resident] had become best friends with Oscar [dog]. He took him for walks, fed him treats, and played ball with him. In return, Oscar spent much of the day with him and slept in his room at night. The resident had a difficult time adjusting to life without Oscar. He felt that this facility should not adopt any more pets. He was afraid to become attached to another pet and then have it removed from the facility (Kendziorski, 1999: 23) |
| Meaningful activity | Although residents acknowledged that they had support from the care environment staff and some volunteers, they wanted to do jobs and be involved in henkeeping through letting the hens out on a morning, looking for eggs, ‘feeding them titbits’. (Cook et al, 2013: 57)  Having hens in the care setting led to purposeful activity occurring within their community. Residents were able to assume roles, such as henkeeper or egg collector, which added meaning to their lives. (Cook et al, 2013: 58)  And they go and feed the fish. We have one customer who goes every morning and feeds them. Yeah, he feeds the fish in the pond…and others come and talk to Joey. (Laughs) ‘Oh hello, Joey, are you a good boy?’ You know. Yeah, it’s really nice. (Care Worker) (Fossey & Lawrence, 2013: 317)  I feed him [Jasper, resident dog] all the time and they get after me because they don’t want him to eat. He’s getting too big. (Marsha, Resident) (Kendziorski, 1999: 32-3)  Several participants expressed their desire for the nursing home to obtain other pets, however, they still stated their physical disabilities would restrict them from participating in the maintenance of the resident animals. (Kendziorski, 1998: 39-40) |
| Caring | Visiting animals |
| Being affectionate and being concerned | One-on-one interaction with the dogs allowed the participants to gradually connect with the dog (“I understand her [the dog] well.”) as well as feel a sense of responsibility for taking part in the dog’s upbringing. They expressed confidence in “having raised the dog until now.” (Kawamura, 2009:44)  Some participants…expressed feelings of ownership and talked with confidence of someone very familiar with the dogs, as expressed in the following quote: “Momo [the dog]…I knew she would settle down in about 10 minutes. Once she settles down…then I understand, ‘Mom is fine’.” (Kawamura, 2009: 44)  I can take them or leave them…I don’t understand you, know, it’s kind of a waste of your time to bring them to us because *we can’t adopt any of them*. (Melissa, Resident) (Roenke & Mulligan, 1998: 35) (reviewer emphasis)  Feelings of tenderness appear when protecting the dog from danger with one’s hands like a shield, holding the dog close in one’s arms for fear of danger that the dog might hurt himself:  The DH [dog handler] takes out a comb to comb the dog, Mrs Andersson grabs the comb from DH and opens her eyes wide, puts the comb aside and looks at DH saying “No, put the comb far away from the dog”. (Mrs Andersson) (Swall et al, 2015: 88)  Mrs Anderson: [Sits up and looks at the dog.] Well, you are so cute . . . Well you are so beautiful. [She bends down towards the dog: You are so beautiful.  You are so beautiful. [Takes a sweet from a plastic bag and gives one to the dog.] You are so beautiful. [The dog eats the sweet.] Was it good? [Bends down towards the dog and looks at him.] Did you like the sweet? [Still looking at the dog. The dog comes forward and puts it head in Mrs Anderson’s lap. She leans against the dog]. Well you are so beautiful…(Swall et al, 2017: 4)  Mr Edgar: Are you limping, my friend? [He looks at the dog]  Dog Handler (DH): Yeah, he has a bit of pain in his front end.  Mr Edgar: OK…[He follows the dog with his eyes. His voice becomes somewhat quieter and darker, and his expression becomes serious]  DH: Do you think it will pass?  Mr Edgar: Yes, I hope so…DH: yes…I think so.  Mr Edgar: Yeah, let’s hope so.  DH: He gets medicine for his limping.  Mr Edgar: Ooh well.  DH: So, the vet thinks so.  Mr Edgar: Yes, yes it appears that he has problems when he walks…Oh, oh poor little chap, yes, yes. (Swall et al, 2017: 5)  Some residents expressed a preference for interacting with a known animal…Yvonne said she prefers to see the same animal more than once “because they get to know me, and they run in and jump on my knee right away”…Cathy pointed out that not being able to develop a relationship with the visiting animal was a barrier to the interaction: “They’re not your dog. It’s going to be a short visit…you’re not going to get emotionally involved with them.” (Wong & Breheny, 2021: 2649) |
| 4. Respite |  |
| From loneliness | Leaving home is a trauma on its own, you’re losing your home, you’re cut off from friends if it’s a distance. And it’s a complete change in your life. And to take away the very thing [the pet] that gives you stability and comfort is lacking in imagination. (Nina, Resident) (Freedman et al, 2021: 1974)  She’s a comfort to me. I had to sell my home and all my possessions. Sasha [her dog] is all I’ve got left. Something is always there with her around. She is my constant companion. (Resident) (Pooley, 2007: 8)  They’re so much company when you’re alone, you know. They sit with you, and you ask them to come and they come…You must believe that when you’re alone and you have an animal, you’re not alone. You have somebody with you. (Alice, Resident) (Roenke & Mulligan, 1998: 35)  Cathy has a sense of clarity of the things that are and are not important to her, and the occasional brief encounter with an unfamiliar animal in the hallway does not feature as an important aspect of her life. What are important are human social encounters; Cathy feels there are limited opportunities for these inside the rest home…Cathy’s longing for “social encounters” is related to the loneliness and social isolation that is often associated with ageing and RAC [residential aged care] for a variety of reasons. (Wong, 2015: 94-5) |
| Building relationships with other residents and staff | It is comical watching them…one was pecking the window and so the hens cause you to laugh a bit and enjoy a bit of fun with the residents. (Care Worker) (Cooke et al, 2013: 61)  Two of the residents normally don’t talk to each other. But when they had the dog between them on the couch, they talked and talked. It is just fantastic to see what a dog can do. (Nurse) (Gundersen & Johannessen, 2018: 107)  She loves him [the dog] and he does her…Every evening, I obviously go with them, they walk right to the end of this garden…You can sit there with him for a while and take in the evening air (laughs). I sit with the two of them every evening. It’s very rare that we don’t…if it’s rained and then it’s stopped we can still walk up there…It’s lovely and it’s good for her. (Jack, Resident) (Freedman et al, 2021: 1971)  Everybody likes him, you know, he goes round to see various people, all the people who make him welcome he visits and he thoroughly enjoys it. (Diana, Resident) (Freedman et al, 2021: 1974)  I suggest to other residents to come to AAA. When they come, then I just watch them with the dogs from beginning to end. (Resident) (Kawamura, 2009: 45)  As the participants’ interactions with the dogs deepened, their interests also began to spread to human participants. The AAA sessions broadened interaction; with this progress in interaction, participants developed an interest in their neighbors and were able to enjoy time with them. (Kawamura, 2009: 46)  Well…we sit…in the lobby together, some of the friends you know…when they bring the bunny in and bring it over to us we’ll play with it. (Resident) (Pitheckoff et al, 2018: 1570)  Nearly all of the pet owners spoke of how their companion animal had helped them to make friends with staff and other residents. Many pet owners spoke of sharing their pet with other residents living in the home. Many pet owners spoke of their pleasure at the care that staff showed towards their pet, which seemed to make the pet owner feel more cared for themselves. (Pooley, 2007: 8-9). |
| Connecting with others | It’s me visiting, and the dog is my tool. Then we can sit and talk about my dog. It’s just as much my presence as the dog’s. (Dog Handler) (Gundersen & Johannessen, 2018: 106)  I talk about the dog and start conversations with them about the dog. An advantage in dementia care is that they quickly forget, so you can repeat it a few times. (Dog Handler) (Gundersen & Johannessen, 2018: 106)  They [the volunteers] are all young people, so I like getting energy from them. They remind me of my younger days, and I remember that I was once like that a long time ago. Because the student volunteers change periodically, we can feel the passing of time. (Resident) (Kawamura, 2009: 45)  We are more like friends and family to the people there, and it is important that they feel free to treat us that way. Some people are shy or reticent when staff are around, and we would not want that to happen with us. (Volunteer) (Savishinsky, 1985: 123)  Interviewer: Um, what do you enjoy the most about the visits?  Andrew: I don’t know if it’s [volunteer’s name] company, or the dogs, or the whole lot, you know. All the visitors, the animals. Yeah I’d say it’s the animals, and [volunteer’s name], you know, communications. (Wong & Breheny, 2021: 2652) |
| From institutionalisation | …henkeeping in care settings added a different positive element to daily life for those living in these environments. These animals added a new and interesting dimension to the community. (Cook et al, 2013: 65)  I think it has brought life to the garden. Before the hens arrived the garden was just a big empty space. Now the doors are always open and residents enjoy being in the garden. When families visit they bring their relative to the garden and they have fun times together. (Care Worker) (Cook et al, 2013: 61-2)  It annoys me when you hear people at breakfast time, lunch time and evening meal, the first thing they’re asked after they’ve finished their meal is shall we take you back to your room? Now what an existence…*Having a pet, it gives you more to life…it’s something new every day to do*. (Jack, Resident) (Freedman et al, 2021: 1973) (reviewer emphasis)  Things that matter a lot, such as not having too many participants and *having the same participants in the visits so that it’s possible to follow up with them* and see the impact it may have on them. (Dog Handler) (Gundersen & Johannessen, 2018: 107) (reviewer emphasis)  The sessions…provided a ‘breath of fresh air’ for the participants, through which they felt relief due to a break in their daily routine. One woman said: “Even if we stay in the same room…we can’t really discuss something with our roommates. We just sit in the same room without saying a word…I feel gloomy, so I go to see the dog.” (Kawamura, 2009: 45)  The comments of the participants indicate that the chief characteristic of the AAA experience was a change from daily life. It is very important for volunteers or people from outside the nursing home to make residents feel refreshed and alleviate their feelings of loneliness or anger related to the restriction of their lives. (Kawamura, 2009: 45)  With a regular pet visitation schedule, residents began to expect the pet to come and looked forward to that day. “The guy who comes with the big white dog, he’s beautiful. He’s great. Somebody said he was coming back again pretty soon. He’s smart that dog…I’ve never seen any dog like that before. Where does that man live? He told us and I can’t remember where. He doesn’t live too far away. He and his dog come often, probably every three months…It’s about time for him to come around…” (Karen, Resident) (Roenke & Mulligan, 1998: 37)  The residents in the nursing homes don’t remember me, but the assisted living homes do know me and look forward to the visits. They are always gathered waiting my arrival. (Beth, Volunteer) (Roenke & Mulligan, 1998: 37)  I love it. I look forward to it…I’d be very upset (if she missed seeing the visiting animals). Because I look forward to seeing them so much. (Yvonne, Resident) (Wong, 2015: 85)  Interviewer: Do you think having the animals come in here makes a difference to [the experience of living here]?  Andrew: Well, no, it doesn’t really. But while they’re here, it gives you a lift. *Because the infrequency*. (Andrew, Resident) (Wong & Breheny, 2021: 2648) (reviewer emphasis)  …And, to what extent am I going to get involved with the animal? I’m going to get to cuddle it, and I - seeing them, they bring particularly nice animals, I see them in passing and always stop, but they have *no significant role in my life at all*…They are just part of a pleasant, yeah definitely a pleasant aspect, but *a very fleeting one*. (Cathy, Resident) (Wong & Breheny, 2021: 2650) (reviewer emphasis) |
| From symptoms of illness | One lady was in the final stage of dementia. She did not communicate vocally at all. She would sit all day and just rock. When the chicks were hatching we gathered the residents around the incubator and as the chicks hatched she started to say ‘chicken chicken…’I was very tearful…she put out her hand and cupped the chick and tried to kiss their beak. That is why I am so enthusiastic about the hens. (Care Worker) (Cooke et al, 2013: 61)  It reminds us even more of how important it is to create a good moment, and that, okay, even if it’s not something that will last, that they will remember for a long time, it’s there in the moment. (Nurse) (Gundersen & Johannssen, 2018: 107)  “So I saw this person who I was going to meet, and asked him, “Would you like to stroke the dog?” This is a man who does not talk...so much**…**he can walk with a walker, but needs the support of two caregivers…and he’s a bit agitated…the dog walks up and puts his head in his lap, and then he releases his clenched hands,…he clenched his hands so tight that his hands were completely red…It is like he’s trying to hold himself together. And so he begins to caress the dog on the head and then he sits there for a while…so I ask him, “Would you like to hold the dog on a leash?” Then he nods but does not say anything ... so we help each other and so we put the leash on the dog. He takes his walker and puts it aside, and then turns around and stands up…and then he walks away with the dog. He walks down the hall, and goes all the way down the hall and the caregivers come running…but they stop themselves, and are surprised he is walking...so we follow him. And so he goes to his room, turns around, sits down on the bed, opens his drawer and takes out a picture of his dog, and so he says to the dog, “This, this is my dog”…(Mary)” (Dog Handler) (Swall et al, 2016: 2228)  Resident A stated four separate times, “you make me feel better, yes, you do.” Resident A reported that she had fallen the night before and hurt her head. During this visit a doctor came in to check on resident A and resident A reported, ‘I feel much better with Hailey [the dog].’ Hailey laid next to resident A who put her arm around Hailey…Resident A’s eyes remained closed, and her mouth opened with a smile that remained on her face. When the visit was over, resident A puckered her lips and gestured a kiss. (Zando, 2017: 18)  Resident C lifted up his head from his pillow to look down at Hailey. He would rest his head for a minute and then lift it up again. Resident C laughed distinctly twice. Resident C gave a thumb up to Hailey after her tricks and a high five to this researcher as she left. (Zando, 2017: 19) |
| Relief from pain and anxiety [at end of life] | …and every time she opened her eyes and saw the dog, she said, ‘So wonderful’…She had a good last night, because otherwise she had a lot of anxiety, but that night was good for her… (Beth, Dog Handler) (Swall et al, 2019: 68)  …it [the dog] does everything from encouraging and stimulating to…just being there…and a common thing in the final stage in life is the suppression of death anxiety… (Ann, Dog Handler) (Swall et al, 2019: 68)  …many talk about their own future death…a man with aphasia, he talked to the dog when he was approaching the end of his life: ‘Soon, I won’t be here anymore’ and he did not look at me; he was talking to the dog the whole time… (Mandy, Dog Handler) (Swall et al, 2019: 67)  The handlers…felt one of the reasons that persons with dementia shared deep, psychological and existential feelings and emotions with the dog, is that the conversation did not feel pressured as they did not have to answer any questions. They also knew that the information they shared with the dog would stay there. (Swall et al, 2019: 67)  …we had a man…he was approaching death…dogs had been a big part of his life…the dog climbed up so that it was within sight…his daughter said: Do you see the dog…? And then he opened his eyes and then he saw the dog…he had a smile all over his face, and then he fell asleep…the third time he woke up and smiled…he started to pat the dog…the physiotherapist and the daughter were shocked…he had not used that arm for a very long time…he died the following morning. (Christine, Dog Handler) (Swall et al, 2019: 68) |
| 5. Sensory engagement |  |
| Physical contact/touching/petting | I think this is something I would highly recommend, especially for the residents with dementia, it was a form of communicating and engaging, you know being able to touch and pet the animal, I found that was quite effective. (Care Worker) (Casey et al, 2018: 1241)  They are holding their arms around him, he sits close to them and they are almost laying on top of him. I see that it gives them a physical contact that they need… They are completely crazy about her. A man without language, only a few sounds, he was cuddling and cuddling. (Dog Handler) (Gundersen & Johannessen, 2018: 105-6)  When the residents petted the rabbits, they often smiled and greeted them. Verbatim quotes included ‘I love bunnies’, ‘I like to pet them’, and ‘You’re a little sweetheart’. (Pitheckoff et al, 2018: 1569)  I believe that the touching and interaction with pets provides the foundation and stimulus for reminiscence. After speaking about their past experiences with pets, I have observed elderly persons remember and talk about childhood events that were unrelated to pets. Such reminiscence is healthy because it places meaning and importance onto one’s life and to ordinary routines and is positive for one’s emotional well-being. (Roenke & Mulligan, 1998: 38)  I get both verbal and non-verbal responses [from residents]. Those residents that are not able to speak or even see very well have a look of contentment when *you place the pet on their lap or put their hand on the animal’s fur*. It’s a wonderful response to see…those that are verbal will sometimes just moan a sigh of contentment and some will ask questions. (Beth, Volunteer) (Roenke & Mulligan, 1998: 39) (reviewer emphasis)  Being aware of the dog and its body, describing how the dog feels with words like: warm, heavy, soft and calm, Brushing the dog’s coat, with a gentle touch, but also uncertain if one is doing it right, sometimes preferring to pat the dog’s coat with the hand rather than with the brush. (Swall et al, 2015: 88)  Well they just come and sit on my knee, cuddle up to me, lick my face. And it’s so nice. (Yvonne, Resident) (Wong, 2015: 85)  …I don’t know, it’s just good…And I think particularly, someone like me who’s been bedridden for a while, someone bringing a puppy or a kitten and letting them snuggle up against you is lovely. (Jean, Resident) (Wong & Breheny, 2021: 2648)  Resident B was sitting in his wheel chair in his room. When resident B saw Hailey [the dog] he smiled and put out his arms to pet her. (Zando, 2017: 20)  Resident A opened her eyes half way and closed them...Resident A slowly opened her eyes again, this time making eye contact with Hailey [the dog]. Resident A instantly opened her eyes wide, mouth opened with a wide smile, and extended her arms straight in front of her. Introductions were made. Resident A was verbal and stated ‘hi sweetie.’ (Zando, 2017:15) |
| Watching | Oh I spend time with them, they are always doing things. I sit and watch them. They come, they go. There is always something to see. (Resident) (Cooke et al, 2013: 55)  The daughter brought him here and her sister couldn’t believe the expression on his face. It made her cry. When I spoke with them I asked what is wrong. She said he thought he was going in a care home and would be in his room all day…now *he watches the hens and enjoys the garden*. They are so happy. (Care Worker) (Cooke et al, 2013: 61) (reviewer emphasis)  It is comical watching them…one was pecking the window and so the hens cause you to laugh a bit and enjoy a bit of fun with the residents. (Care Worker) (Cooke et al, 2013: 61)  They’re fond of the fish. They like the colourfulness, they like the colours of the fish and they go on and see what fish we have here. Yeah they are aware of them. (Care Worker) (Fossey & Lawrence, 2013: 317-8) |
| Pleasure and joy | Anybody that came in contact with the animals at the time were very happy. I didn’t see or hear any negativity at all. (Care Worker) (Casey et al, 2018: 1241)  Residents really enjoy having the hens here (Care Worker) (Cooke et al, 2013: 61)  Staff spoke with certainty about the pleasure that individuals derived from interacting with a wide range of animals, often recounting their surprise at the positive response that this elicited in residents in advanced, as well as earlier stages of dementia. (Fossey & Lawrence, 2013: 316)  I see an incredible joy. Several times when we have come, one of the residents has been sad, depressed…but once we are there, everything is okay…When they see the dog, most of them become happy and are smiling. (Gundersen & Johannessen, 2018: 105)  …oh, it just gives me the loveliest feeling. I just adore them. And I say. ‘Well, come on, give me a kiss,’ and I’ll get licked all over. (Annie, Resident) (Wong, 2015: 116) |
| Calmness | Hens have a calming effect – they [the residents] relax when watching the hens. (Care Worker) (Cook et al, 2013: 61)  One resident who is walking around a lot, often in a bad mood, sat calmly during the visit, smiling and laughing. (Nurse) (Gundersen & Johannsessen, 2018: 107)  When the dog sits next to them and they can get close to it, they sit quietly and are relaxed. It looks as if they get an inner calmness, in a way. (Dog Handler) (Gundersen & Johannsessen, 2018: 106)  Ms A…stated how petting their fur kept her from repeatedly scratching her arms: ‘It calms me down…I rub their fur and it relaxes my mind’. Another stated ‘Calm…satisfied…comforting’ when asked how he felt when petting the rabbits. (Pitheckoff et al, 2018: 1569)  The findings show the participants…to be calm. They expressed sounds of contentment and almost fell asleep when caressing and being close to the dog. (Swall et al, 2017: 5) |
| 6. Staff |  |
| Enhanced workplace | …Everybody that I noticed was so much more pleasant, you know, smiling when the animals arrived. They were just, they’re so cute. [laughs] (Care Worker) (Casey et al, 2018: 1242)  Staff throughout the whole building were like ‘oh my goodness the animals are here’ and they come poke their head in and every single staff member that came in here *had a smile on their face when they saw the animals*…they just wanted to come in and pet the goat, just take time out of their own day to come in and see how things were going. (Care Worker) (Casey et al, 2018: 1241) (reviewer emphasis)  The AAI initiative was reported to have a positive impact on workload for day staff as it provided more structure that seemed to help enable relief for staff to focus more on providing quality programming for all their residents. (Casey et al, 2018: 1243)  It did make our jobs easier on the floor. (Care Worker) (Casey et al, 2018: 1242)  I know they’re safe when they’re outside with the animals or in here with the animals. You know, if there’s four or six people of them at a time then there’s four or six people that I’m not looking out for out there on the floor! (Care Worker) (Casey et al, 1243) |
| Seeing residents engage | …it was good for both the staff and the resident to have something totally different from a regular line of duty to do. And it really did have staff just smiling and just feeling good overall, knowing the resident, that it’s making a difference in the resident at that time…it was a good thing for the home, for the residents, for the staff. (Care Worker) (Casey et al, 2018: 1243)  Seeing them happy is nice. (Care Worker) (Casey et al, 2018: 1242)  Day staff…unanimously valued the program and stated that it contributed to a positive feeling within the unit and throughout the institution. These staff reported that the program was beneficial and supported a continuation of the program. Repeatedly, they noted that there was a decrease in agitation and aggressiveness among residents on AAI program days. They also reported that it was a relief to observe these residents were more content even though they note there was not a long term impact on behavior or affect. (Casey et al, 1244)  A certain resident hadn’t been speaking and you should have seen the difference in this person with this dog. It was unbelievable the dog. (Care Worker) (Fossey & Lawrence, 2013: 316)  They enjoyed it, because some of them have always had animals. I’ve always had animals, I mean I have always had a dog, and I think if I ever get old and got put in a care home I wouldn’t go unless they let me have my dog. (Care Worker) (Fossey & Lawrence, 2013: 317)  I can’t go on enough about the role of animals in this sort of environment. It is tremendous. (Manager) (Pooley, 2007: 9) |
| Better caring culture | One issue that was frequently raised…was the negative impact that communal animals could have on particular residents who were at risk of being disturbed or distressed by their presence. (Fossey & Lawrence, 2013: 324-5)  …his volunteering is very much appreciated, because we see what it does with the residents and the joy it gives them. (Nurse) (Gundersen & Johannessen, 2018: 108)  The dog handlers…had the skills necessary to design visits that met each person’s needs, which might have decreased the burden of the caregivers in surrounding wards during and after the visits. This may contribute towards a more home-like environment, and may in turn create a better caring climate that induces person-centredness and might increase well-being for both the caregiver and the person with dementia. (Swall et al, 2016: 2229) |
| Increase workload | Having the HENPOWER programme…added to the workload of staff. Staff worked to fit this additional activity within their routines – the positive impact of the hens on the residents’ quality of life became a strong motivator to achieving this within the context of their own care environment. (Cooke et al, 2013: 65)  I thought that we would have to do more for the hens such as cleaning out all of the time. I thought that they were going to be a bit more hard work. I thought that they would smell. But they don’t bother anybody, they just wonder around…I just thought that I would hear them and they would be noisy, but you don’t. (Care Worker) (Cook et al, 2013: 64)  I’m quite happy with people bringing pets in, it’s just as long as they take them out again…because it’s almost like having another resident or two in the home when you have pets in the home…you’ve got their whole care plan. (Care Worker) (Fossey & Lawrence, 2013: 319)  …some participants disliked or were apprehensive about the additional work that caring for communal animals could entail. Staff listed obligations such as feeding, cleaning and exercising animals which were often seen to involve unpleasant jobs such as going outside in the cold or removing animal dropping s from fish tanks or cages. (Fossey & Lawrence, 2013: 323)  …the suitability of an animal was primarily assessed in terms of the level of care that it required. Animals perceived as having clean habits or those that were perceived to be ‘low maintenance’, such as caged birds or cats, were considered preferable. (Fossey & Lawrence, 2013: 323)  The rabbits…because they were outside, so you had to get residents outside and take them inside and staff had to go and clean them, and clean the cage and only certain staff would do it and [mimicking a colleague] ‘I don’t really like rabbits’ so…it became a bit of a problem…and then once, some time they had forgotten, and they weren’t fed. (Care Worker) (Fossey & Lawrence, 2013: 324) |
| Distraction from care | …others suggested that the increased workload would distract them from their prime responsibility of caring for residents. (Cook et al, 2013: 62)  Some…staff argued that caring for animals was ‘not their job’ and could…distract from the care of residents. Participants joked that resident animals required their own care plan and as such placed unreasonable demands on their time. (Fossey & Lawrence, 2013: 324) |
| Health, hygiene and safety concerns | They [staff] were worried that the hens would smell and that there would be mess throughout the home. Others were concerned that the hens would attract vermin to the home. (Cook et al, 2013: 62)  There was one lady…we couldn’t take the dog down that end because she had an allergy. So that was fine…you just accommodate for that, you know, so there’s no problem. (Care Worker) (Fossey & Lawrence, 2013: 321)  …concerns about ‘health and safety’ were frequently cited…yet were poorly defined. This was often presented as a default response that negated the need to consider the topic further. (Fossey & Lawrence, 2013: 322) |
| Management support and organisation | It won’t be successful if the manager isn’t on board…the manager was just against it and she really actively, openly said ‘I don’t like hens, I won’t be involved with the project’. It was then not possible for us to be involved. (Care Worker) (Cook et al, 2013: 64)  And they are so risk averse they just won’t try anything challenging. They don’t consider that having an environment that is not stimulating has a negative effect on resident’s mental health. (Manager) (Cook et al, 2013: 63)  You do not do anything until you’ve thought out every aspect of it. You do not come along and say that I am going to do this until you have thought out every single aspect of that. (Care Worker) (Fossey & Lawrence, 2013: 323)  The nurses also emphasized the importance of an inspiring and supportive leader for the existence of dog visits, The leader had inspired them by explaining the potential positive benefits of dog visits, and advocated for their residents to have this opportunity. “We are inspired by our leader - she gave us information and good feeling about this”. (Nurse) (Gundersen & Johannessen, 2018: 108)  New responsibilities concerning the feeding and cleaning of the pet must be carefully delineated, and the administration must budget for pet supplies and health maintenance costs. (Kongable et al, 1990) |
| 7. Animal  Positive | If they noticed that the dog became uncomfortable, they stopped the session earlier than intended. According to the dog handlers, all the dogs seemed to appreciate the visits. They explained how the dogs were committed to the task and were well-prepared in advance (e.g. getting washed, combed and putting on a Red Cross scarf). A wagging tail showed that they were eager ‘to go to work’. “She (the dog) gets so happy when she knows where we are going, and she behaves so properly in the nursing home. She’s so calm and careful. She knows what to do.” (Dog Handler) (Gundersen & Johannessen, 2018: 106)  One woman…[told] the story of how severely depressed and withdrawn her dog, George, had become ‘after the death of his sister’ in an automobile accident. A few months later, when she began to bring him to the pet program, the sociable interaction with people and animals had helped to draw him out again’. The moral that the woman drew from her story had a familial theme: people are not alone in suffering loss, for animals experience the same devastation when, as she put it, ‘their kin die’. In her vie, the re-engagement offered by pet sessions can be as therapeutic for the animals as for the people they visit. (Savishinky, 1985: 122)  The dog’s closeness makes one realize that the dog appreciates affection when being stroked since it stays close and falls asleep in one’s presence. (Swall et al, 2017: 4) |
| Negative | …risks to the animals because of unintended mishandling by residents due to their diminished cognitive capacities (Casey et al, 2018: 1245)  The rabbits were a whole issue weren’t they…because they were outside, so you had to get residents outside and take them inside and staff had to go and clean them, and clean the cage and only certain staff would do it and [mimicking a colleague] ‘I don’t really like rabbits’, so those things, so it became a bit of a problem…and then once, some time they had forgotten, and they weren’t fed.” (Fossey & Lawrence, 2013: 324)  Some clients kicked at the air in a teasing manner toward the dog, while others grabbed the dog by the tail and attempted to carry it. (Kongable et al, 1990)  An example of a potential safety hazard to the pet is residents grabbing at its collar, tugging its tail, and kicking it, producing a stress overload for the animal. An actual instance described by a staff nurse involved one of the Alzheimer’s residents becoming too possessive with the dog and attempting to pick it up so as to keep it away from the other patients. (Kongable et al, 1990) |

**Figure S1: Thematic Network**

**
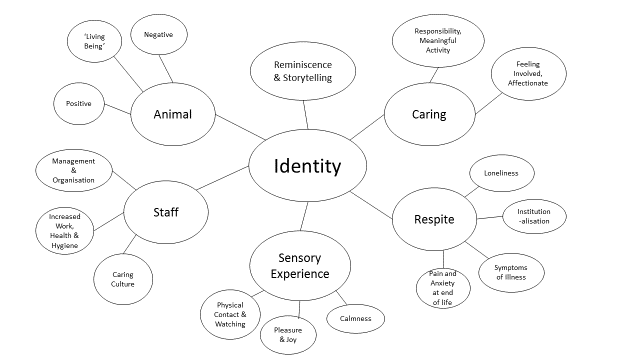
**

**Figure S2: Meta-analyses showing effects of animal assisted intervention on outcomes of depression, anxiety, agitation and quality of life**

Depression


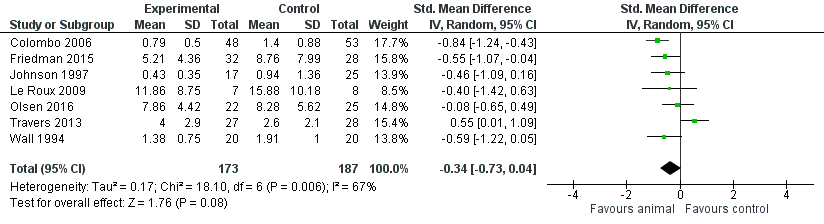


Anxiety


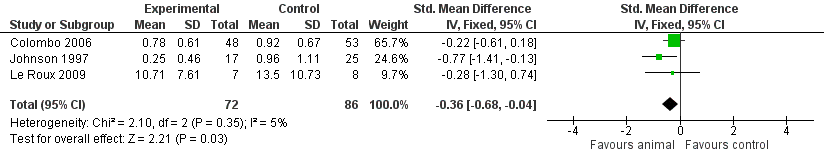


Agitation


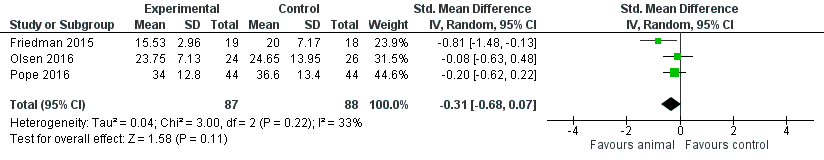


Quality of Life


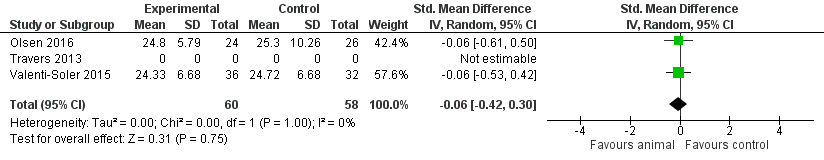


File S2: PRISMA Checklist

| **Section and Topic** | **Item #** | **Checklist item** | **Location where item is reported** |
| --- | --- | --- | --- |
| **TITLE** | | |  |
| Title | 1 | Identify the report as a systematic review. | 1 |
| **ABSTRACT** | | |  |
| Abstract | 2 | See the PRISMA 2020 for Abstracts checklist. | YES |
| **INTRODUCTION** | | |  |
| Rationale | 3 | Describe the rationale for the review in the context of existing knowledge. | 2 |
| Objectives | 4 | Provide an explicit statement of the objective(s) or question(s) the review addresses. | 3 |
| **METHODS** | | |  |
| Eligibility criteria | 5 | Specify the inclusion and exclusion criteria for the review and how studies were grouped for the syntheses. | 5 |
| Information sources | 6 | Specify all databases, registers, websites, organisations, reference lists and other sources searched or consulted to identify studies. Specify the date when each source was last searched or consulted. | 4 |
| Search strategy | 7 | Present the full search strategies for all databases, registers and websites, including any filters and limits used. | Additional file 9medline) |
| Selection process | 8 | Specify the methods used to decide whether a study met the inclusion criteria of the review, including how many reviewers screened each record and each report retrieved, whether they worked independently, and if applicable, details of automation tools used in the process. | 5 |
| Data collection process | 9 | Specify the methods used to collect data from reports, including how many reviewers collected data from each report, whether they worked independently, any processes for obtaining or confirming data from study investigators, and if applicable, details of automation tools used in the process. | 5 |
| Data items | 10a | List and define all outcomes for which data were sought. Specify whether all results that were compatible with each outcome domain in each study were sought (e.g. for all measures, time points, analyses), and if not, the methods used to decide which results to collect. | 5 |
|  | 10b | List and define all other variables for which data were sought (e.g. participant and intervention characteristics, funding sources). Describe any assumptions made about any missing or unclear information. | 5 |
| Study risk of bias assessment | 11 | Specify the methods used to assess risk of bias in the included studies, including details of the tool(s) used, how many reviewers assessed each study and whether they worked independently, and if applicable, details of automation tools used in the process. | 5 |
| Effect measures | 12 | Specify for each outcome the effect measure(s) (e.g. risk ratio, mean difference) used in the synthesis or presentation of results. | 6 |
| Synthesis methods | 13a | Describe the processes used to decide which studies were eligible for each synthesis (e.g. tabulating the study intervention characteristics and comparing against the planned groups for each synthesis (item #5)). | 6-7 |
|  | 13b | Describe any methods required to prepare the data for presentation or synthesis, such as handling of missing summary statistics, or data conversions. | 6-7 |
|  | 13c | Describe any methods used to tabulate or visually display results of individual studies and syntheses. | 6-7 |
|  | 13d | Describe any methods used to synthesize results and provide a rationale for the choice(s). If meta-analysis was performed, describe the model(s), method(s) to identify the presence and extent of statistical heterogeneity, and software package(s) used. | 6-7 |
|  | 13e | Describe any methods used to explore possible causes of heterogeneity among study results (e.g. subgroup analysis, meta-regression). | NA |
|  | 13f | Describe any sensitivity analyses conducted to assess robustness of the synthesized results. | NA |
| Reporting bias assessment | 14 | Describe any methods used to assess risk of bias due to missing results in a synthesis (arising from reporting biases). | Not done |
| Certainty assessment | 15 | Describe any methods used to assess certainty (or confidence) in the body of evidence for an outcome. | Not done |
| **RESULTS** | | |  |
| Study selection | 16a | Describe the results of the search and selection process, from the number of records identified in the search to the number of studies included in the review, ideally using a flow diagram. | 7 |
|  | 16b | Cite studies that might appear to meet the inclusion criteria, but which were excluded, and explain why they were excluded. | NR |
| Study characteristics | 17 | Cite each included study and present its characteristics. | Table 1 |
| Risk of bias in studies | 18 | Present assessments of risk of bias for each included study. | Additional file |
| Results of individual studies | 19 | For all outcomes, present, for each study: (a) summary statistics for each group (where appropriate) and (b) an effect estimate and its precision (e.g. confidence/credible interval), ideally using structured tables or plots. | Table 2 |
| Results of syntheses | 20a | For each synthesis, briefly summarise the characteristics and risk of bias among contributing studies. | 17-19 |
|  | 20b | Present results of all statistical syntheses conducted. If meta-analysis was done, present for each the summary estimate and its precision (e.g. confidence/credible interval) and measures of statistical heterogeneity. If comparing groups, describe the direction of the effect. | 17-19 |
|  | 20c | Present results of all investigations of possible causes of heterogeneity among study results. | Not done |
|  | 20d | Present results of all sensitivity analyses conducted to assess the robustness of the synthesized results. | Not done |
| Reporting biases | 21 | Present assessments of risk of bias due to missing results (arising from reporting biases) for each synthesis assessed. | Not done |
| Certainty of evidence | 22 | Present assessments of certainty (or confidence) in the body of evidence for each outcome assessed. | Not done |
| **DISCUSSION** | | |  |
| Discussion | 23a | Provide a general interpretation of the results in the context of other evidence. | 20-21 |
|  | 23b | Discuss any limitations of the evidence included in the review. | 25-26 |
|  | 23c | Discuss any limitations of the review processes used. | 25-26 |
|  | 23d | Discuss implications of the results for practice, policy, and future research. | 26 |
| **OTHER INFORMATION** | | |  |
| Registration and protocol | 24a | Provide registration information for the review, including register name and registration number, or state that the review was not registered. | 4 |
|  | 24b | Indicate where the review protocol can be accessed, or state that a protocol was not prepared. | 4 |
|  | 24c | Describe and explain any amendments to information provided at registration or in the protocol. | NA |
| Support | 25 | Describe sources of financial or non-financial support for the review, and the role of the funders or sponsors in the review. | 28 |
| Competing interests | 26 | Declare any competing interests of review authors. | 28 |
| Availability of data, code and other materials | 27 | Report which of the following are publicly available and where they can be found: template data collection forms; data extracted from included studies; data used for all analyses; analytic code; any other materials used in the review. | NA |

*From:*  Page MJ, McKenzie JE, Bossuyt PM, Boutron I, Hoffmann TC, Mulrow CD, et al. The PRISMA 2020 statement: an updated guideline for reporting systematic reviews. BMJ 2021;372:n71. doi: 10.1136/bmj.n71

For more information, visit: http://www.prisma-statement.org
